# Analysis and nomograph development for a leaky pipeline carrying plug flow based on numerical modeling and experimental validation

**DOI:** 10.1038/s41598-026-36759-w

**Published:** 2026-03-04

**Authors:** Hicham Ferroudji, Abinash Barooah, Ibrahim Hassan, Ahmad K. Sleiti, Sina Rezaei Gomari, Matthew Hamilton, Mohammad Azizur Rahman

**Affiliations:** 1 Laboratory of Petroleum Equipment’s Reliability and Materials, Hydrocarbons and Chemistry Faculty, Boumerdes University, Boumerdes, Algeria; 2 College of Science and Engineering (CSE), Hamad Bin Khalifa University (HBKU), Doha, Qatar; 3 Mechanical Engineering Department, Texas A&M University at Qatar, Doha, Qatar; 4 College of Engineering, Qatar University, Doha, Qatar; 5 School of Computing, Engineering and Digital Technologies, Teesside University, Middlesbrough, UK; 6 Department of Computer Science Engineering, Memorial University, Newfoundland, Canada

**Keywords:** Leak detection, Multiphase plug flow, Numerical modeling, Statistics, Wavelet transform (WT), Non-dimensionless analysis., Energy science and technology, Engineering, Mathematics and computing

## Abstract

Leak detection in pipelines transporting gas–liquid multiphase flow remains a challenging task due to complex and inherently unsteady flow behavior, particularly under intermittent regimes such as plug flow. Conventional leak detection techniques, which are well established for single-phase flow, often suffer from reduced sensitivity and false alarms when applied to multiphase systems. Motivated by these limitations, the present study investigates leakage characteristics in a horizontal pipeline conveying gas–liquid plug flow under underwater conditions. A three-dimensional transient numerical model based on the Volume of Fluid (VOF) approach is developed to simulate various leakage scenarios, including different discharge sizes and gas–liquid superficial velocities. The numerical results are validated against experimental data obtained from a dedicated multiphase flow loop. Time-series pressure signals are analyzed using statistical metrics, probability density functions, and continuous wavelet transform techniques to assess their effectiveness in identifying leakage occurrence. Furthermore, a non-dimensional analysis is employed to develop a nomograph for estimating gas release velocity from underwater leaks. The results demonstrate that leakage significantly alters pressure fluctuation characteristics and gas void fraction distribution, with detectability strongly influenced by flow conditions and discharge size. The proposed nomograph predicts gas release velocity with reasonable agreement relative to experimental measurements, highlighting its potential applicability for subsea leak assessment and process safety analysis in multiphase pipeline systems.

**Supplementary Information:**

The online version contains supplementary material available at 10.1038/s41598-026-36759-w.

## Introduction

Multiphase flow in pipelines, defined by the concurrent movement of gas, liquid, and sometimes solid phases, is a vital occurrence in several industrial applications, including petroleum, chemical, and process engineering. An essential element of multiphase flow analysis is comprehending the pressure drop response within a pipeline under various circumstances, including leakage occurrence. The latter can occur due to multiple causes, including corrosion phenomena^1^, pressure surges like hydraulic hammer and pressure pulsations^2^, Material Defects^3^, in addition to other factors related to infrastructure aging, multiphase flow dynamics, and thermal stresses.

Leakage in a pipeline carrying multiphase flow can be easily identified using simplified methods under some flow conditions, such as stratified flow. However, it can be a challenging task when it comes to intermittent flow, which results in false alarms^4^. This can be explained by the fact that stratified flow presnts favorable conditions for leak identification owing to its stable phase separation. In contrast, slug flow is characterized by severe velocity and pressure oscillations. These intrinsic fluctuations significantly hinder the identification of leak signatures resulting in false alarms. The flow pattern in two-phase flows, both upstream and downstream of a leak, significantly impacts the pressure gradient and influences the leak’s location^5^. Leak detection in multiphase pipelines remains a substantial issue owing to complicated flow regimes and equipment limitations^6^. Recent advancements encompass a wireless sensor network system for precise leak localization and rate determination^7^. For instance, the fuzzy classification of time-domain characteristics derived from flow rate, pressure, and temperature data has shown potential in differentiating leaks from operational variations^8^. For chronic leak detection, ratio of sequential probability testing employing real-time transient monitoring has proven its effectiveness in multiphase flows, while distributed sensing technologies utilizing fiber optic cables present opportunities but encounter challenges related to installation and operational risks^9^. Despite advancements, further analysis is essential, especially concerning subsea and arctic environments, considering time-series data collected from sensors installed on pipelines^9,6^. Under subsea and Arctic conditions, sensor deployment is significantly constrained by the severe operating environment. For instance, low temperatures, extreme hydrostatic pressure, low temperatures, corrosion, and limited accessibility increase installation and maintenance costs while reducing long-term reliability. As a result, pipeline operators are often reluctant to install sensors directly beneath sea level. Instead, monitoring strategies frequently rely on sparsely distributed sensors or indirect leak detection approaches, as can be found in several subsea and Arctic field deployments reported in the literature^10,11^.

### Multiphase flow in pipelines focusing on numerical modeling

The dynamics of two-phase fluid flow, including liquid and gas phases, in a horizontal pipeline is a complicated phenomena that has been thoroughly examined owing to its significance in several sectors, including power generation, chemical processing, and oil and gas. Pressure fluctuations inside the pipeline are essential for comprehending and forecasting the functioning of these systems^12^. A primary component affecting pressure changes is the flow pattern, which delineates the distribution of liquid and gas phases inside the pipeline. The flow pattern may vary from bubbly flow, characterized by the gas phase distributed as tiny bubbles within the liquid, to stratified flow, where gravity separates the gas and liquid phases. The flow pattern is governed by elements like fluid characteristics, flow rates, and pipe geometry^13^.

Numerous researchers have suggested methodologies to estimate the pressure drop in a multiphase flow system, considering various approaches, including numerical modeling, experimental testing, and mechanistic methods. These approaches often associate pressure-drop calculations with the anticipated flow pattern since the pressure drop is significantly influenced by the distribution of phases inside a pipeline^14^. Two-dimensional numerical simulations were conducted by Thaker and Banerjee (2013) to examine the transition from stratified flow to slug flow and the evolution of slug, plug, and annular flows. The method of Volume of Fluid (VOF) was used by Nasrfard et al.^15^ to delineate the liquid-gas interface and its progression over time. The proposed computational fluid dynamics (CFD) model effectively predicted the development and propagation of the intermittent flow in a horizontal circular pipe. Andrianto et al.^16^ focused on the comparison of numerical results obtained from commercial CFD software (Ansys-Fluent) and experimental data of liquid-gas two-phase flow through a horizontal pipeline. The authors considered the liquid holdup parameter for comparison purposes, where a satisfactory matching was reported between the two approaches.

Pineda-Pérez et al.^17^ investigated highly viscous two-phase intermittent flow using the VOF technique, focusing on velocity distribution and liquid volume fraction. A parametric study of several parameters, such as angle factor, sharpening factor, and interface momentum dissipation model was conducted. After that, the authors carried out a comparison of numerical results with experimental findings obtained from an experimental setup. Subsequently, the authors extended their CFD model to consider more ranges of operating conditions. Ban et al.^18^ numerically modeled two-phase flow (oil-gas) in horizontal pipeline to analyze the evolution of associated slug frequency, pressure drop, and liquid holdup. The authors validated their results with Baker’s flow regime chart^19^ and proposed correlations for liquid holdup.

The Volume of Fluid (VOF) approach has been widely used to model liquid-gas two-phase flows due to its accuracy in representing dynamic gas-liquid interfaces and yielding insights into pressure drops, liquid holdup, and slug frequency^18,20^. These investigations have shown strong concordance with experimental data and established relationships. These numerical methods have proven effective in precisely modeling intricate flow regimes in horizontal pipelines, offering significant insights for pipeline design and operation across numerous sectors, including oil and gas production and transportation.

### Multiphase flow in pipelines with leaks

Multiphase flow in pipelines with leaks is a critical study area, particularly for ensuring safe and efficient energy transportation. Various approaches, such as computational fluid dynamics (CFD) simulations and experimental analysis, have been considered to understand flow characteristics and improve leak detection. Experimental investigations have notably progressed in delineating leakage behavior in liquid-gas two-phase flow pipelines, especially via flow loop experiments^21–23^, highlighting the pivotal influence of flow regimes, leak dimensions, and detection methodologies including acoustic techniques^24–27^ and time-series signal processing^28,29^.

Since leakage phenomena in multiphase flow systems is mainly influenced by the flow regime occurring within the pipeline, some researchers conducted experimental analyses taking into account various flow regimes. It was found that, for stratified flow, gas-dominant leakage is prevalent for top leaks, but mixed-phase leakage can arise under high pressure^26^.

In the last decade, advanced techniques have been incorporated into experimental investigations, like signal processing methods^30,29^, which greatly improved leakage detection accuracy. Moreover, acoustic and pressure-based methodologies started to be promising tools to address challenges encountered in transient two-phase flows^25,11^.

Recently, Meng et al.^23^ performed an experimental study regarding the impact of leakage on multiphase flow encompassing leakage mass flow rate, pressure drop, phase separation, and slug frequency inside a horizontal pipeline, considering leakage in various positions. The Standard Deviation (SD), Probability Density Function (PDF), Cumulative Probability Density Function (CPDF), and Power Spectral Density (PSD) methods were employed to analyze their experimental data. Another experimental work conducted by Ferroudji et al.^21^ about the impact of different leak scenarios (none, one, and three leaks) on the behavior of a multiphase flow system (liquid-gas), emphasizing pressure signals captured by dynamic pressure sensors positioned in the upstream and downstream areas. Pressure drop fluctuation coefficient, Probability Density Function (PDF), Cumulative Probability Density Function (CPDF), and Wavelet Transform (WT) were used for the analysis of the collected signals. Additionally, the influence of leaks on the flow-regime map was evaluated. This analysis was enhanced by using several Machine Learning (ML) models to forecast leak occurrences.

Bueno et al.^31^ carried out a numerical model of stratified two-phase flow in a nearly horizontal pipeline with a leakage where they focused on the impact of leak diameter and location on pressure drop. Based on the Euler-Euler model, Tavares et al.^32^ used CFD-based simulations to address leakage in liquid-gas two-phase flow in horizontal pipelines. Analysis of pressure, volume fraction, and velocity were included and discussed in their study. Adegboye et al.^33^ performed numerical modeling to examine the behavior of liquid-gas two-phase flow with leakage in a subsea natural gas pipeline. The simulation findings have been corroborated with the most recent experimental and numerical data documented in the literature, yielding a strong concordance. The impact of leak dimensions, multiple leak occurrences, and axial leak placements on the pressure drop, flow rate, and volume fractions throughout the pipeline were methodically examined.

Ferroudji et al.^24^ developed a 3D numerical model based on the Volume Of Fluid (VOF) method in conjunction with the *k*−*ε* turbulence model to examine the simultaneous leakage occurrence (first leak 3 mm and second leak 1.8 mm) in a pipeline transporting a multiphase flow. The numerical outputs are corroborated by experimental data obtained from a laboratory flow loop system, revealing a substantial degree of coincidence. Furthermore, the flow dynamics inside the pipeline and the adjacent regions of the leaks (water tank) were assessed. Ren et al.^34^ numerically analyzed a leaky pipeline transporting multiphase flow under plug flow conditions. The authors focused on the influence of liquid and gas superficial velocities and leak axial position on the leakage flow and bubble shape by comparing both cases of leaky and non-leaky pipelines.

In terms of numerical modeling, most of the previous studies focused on stratified flow due to its simplicity compared to intermittent flow. For that reason, more attention needs to be paid to leakage modeling in pipelines conveying multiphase flows under intermittent flows, particularly with the enhancement of the treatment and storage capacity of super-calculators. Besides, numerical modeling of the surrounding area of a leaky pipeline conveying multiphase flow also needs to be addressed to understand more about the dispersion phenomena in subsea pipelines. On the other hand, due to the main differences in the modeling approaches between OLGA (1-D modeling) and CFD softwares based on the Finite Volume Method (3-D modeling), the evolution of two-phase flow with leakage over time remains inadequately addressed, particularly for intermittent flow conditions.

In the broader context of pipeline integrity management, data-driven and machine learning approaches have been increasingly applied to account for operational degradation mechanisms such as corrosion, metal loss, and aging. For example, recurrent neural network (RNN)-based frameworks have been developed to estimate pipeline life and classify metal loss dimensions even in the presence of missing input parameters, demonstrating high predictive performance for dry gas pipeline health assessment using historical monitoring data and condition indicators^35^. Similarly, hybrid artificial intelligence models that integrate statistical knowledge and neural networks have been proposed for predicting burst pressure of corroded pipelines, achieving low mean absolute percentage errors and high coefficients of determination, thereby improving the reliability of failure pressure prediction under varying defect conditions^36^. Furthermore, machine learning has been applied to predict corrosion rates of structural steel in operational environments, enabling more accurate estimation of degradation progression and supporting proactive integrity management in soil or subsea conditions (Dong et al., 2024). These studies exemplify the growing use of data-driven methods in structural integrity assessment, which can complement physical leakage analyses by incorporating degradation effects and operational variability.

This work addresses the aforementioned gaps concerning the leakage characteristics of two-phase plug flow in a horizontal pipeline, considering both the internal and surrounding regions. In the first stage, a time series of pressure drop signals are recorded in the upstream and downstream parts for various values of gas and liquid superficial velocities, considering non-leaky and leaky cases. After that, a statistical analysis is performed through the determination of the standard deviation, Probability Density Function (PDF), and dimensionless methods. Moreover, a nomograph was established to predict leakage release velocity in the case of underwater leakage occurrence.

## Methodology

**Fig. 1:**
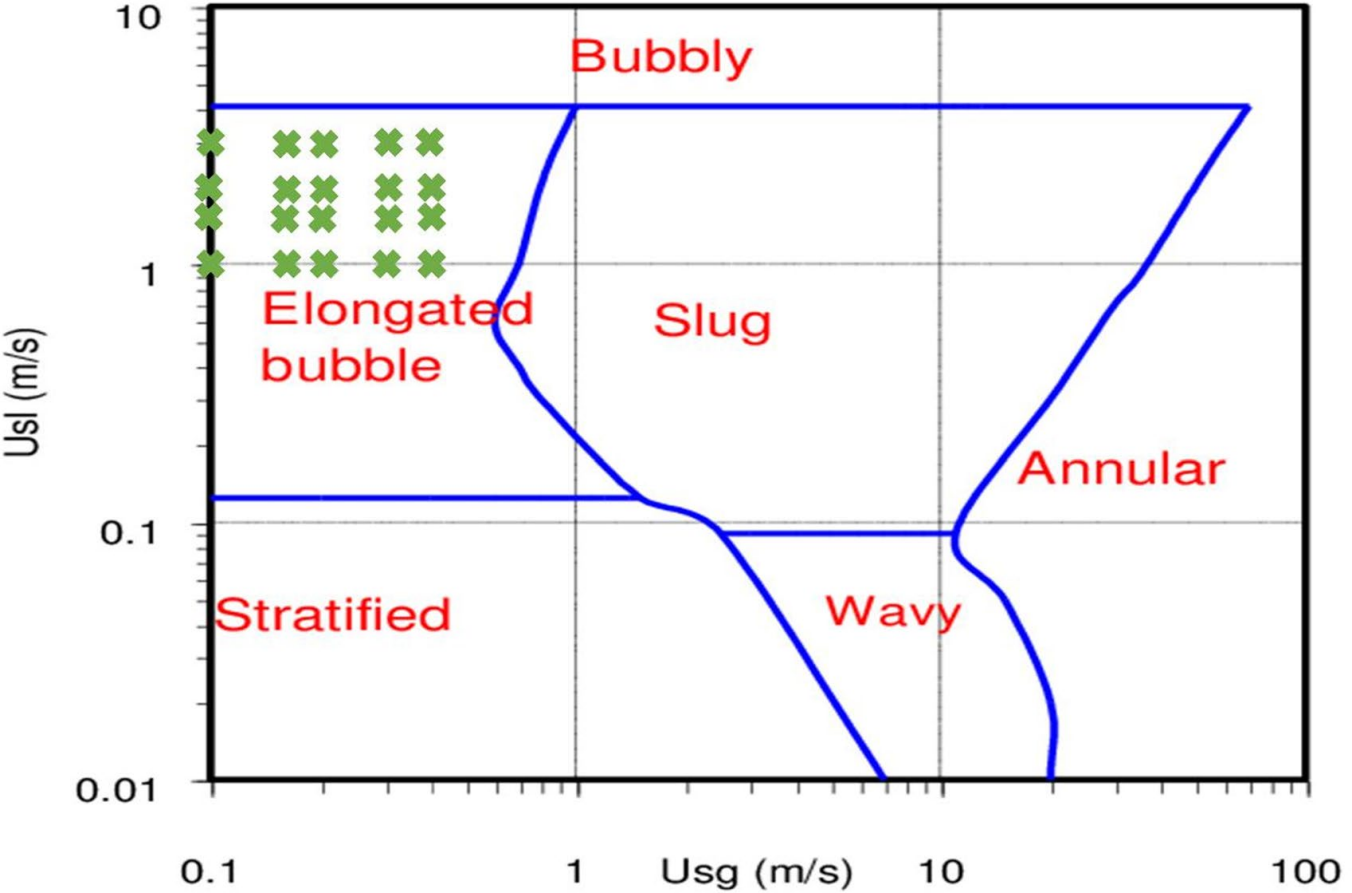
Operating conditions of gas and liquid superficial velocities on the flow regime map of Mandhane et al.^37^.

The current investigation focuses on the impact of discharge (leakage) occurrence in a horizontal pipeline carrying gas-liquid two phase flow (plug flow) considering underwater conditions to mimic offshore pipelines. In addition to the flow analysis within the pipeline, the investigation includes the flow behavior around the discharge area by considering a water tank to visualize gas flow dispersion. Moreover, since the plug flow is considered, the operating conditions of gas and liquid superficial velocities varied within the ranges of 0.1–0.4 m/s and 1–3 m/s, respectively. Figure 1 highlights the considered cases on the flow regime map of Mandhane et al.^37^ for a horizontal pipeline.

### Geometry generation

**Fig. 2:**
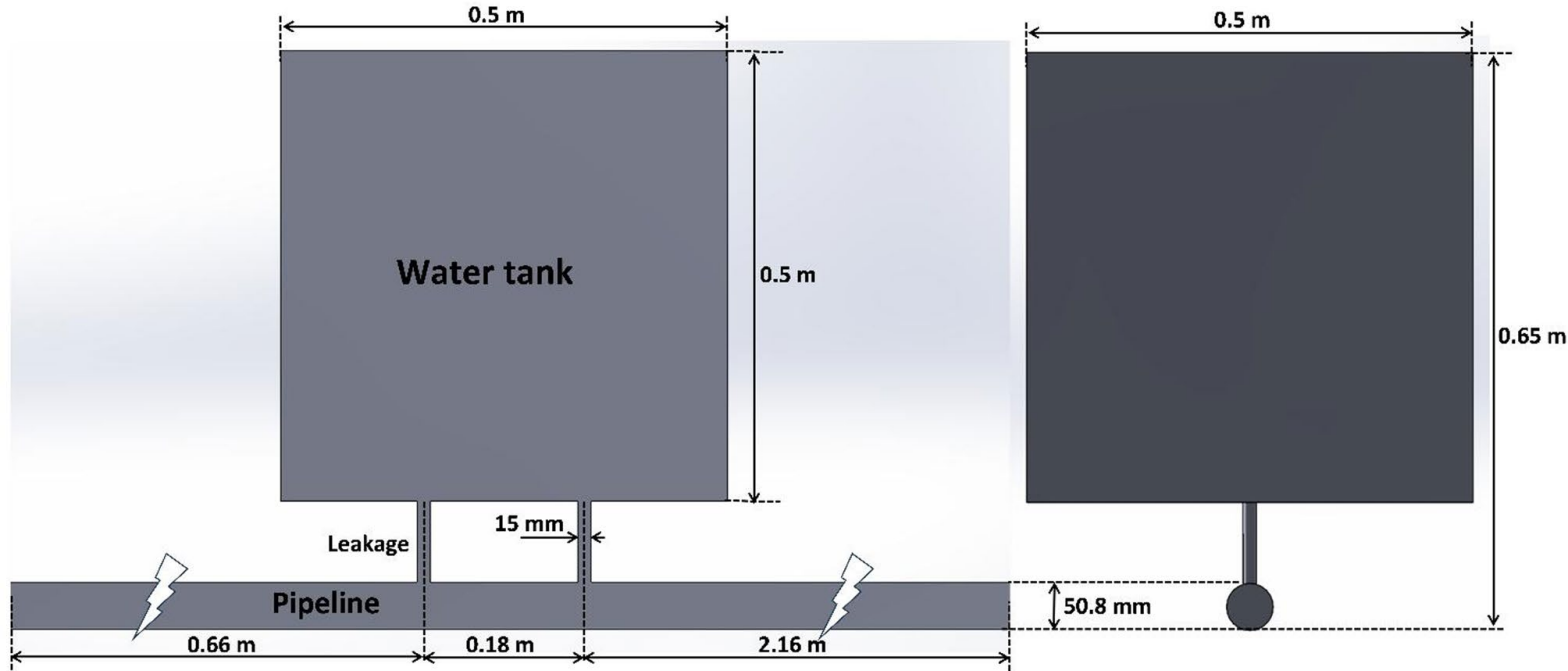
Geometric characteristics of the flow domain for the case of double-leakage (15 mm).

The generated geometry via computer-aided design software (SolidWorks) is consistent with the experimental setup for validation purposes. Initially, a pipe with a diameter of 50.8 mm is created for the multiphase flow domain. After that, several cases were considered, including single-leakage and double-leakage, as well as no-leakage, as a reference case for comparison reasons. Additionally, a water tank (0.5 × 0.5 × 0.5 m) is connected to the main pipeline through leakages, as can be observed in Fig. 2.

### Mesh generation

**Fig. 3:**
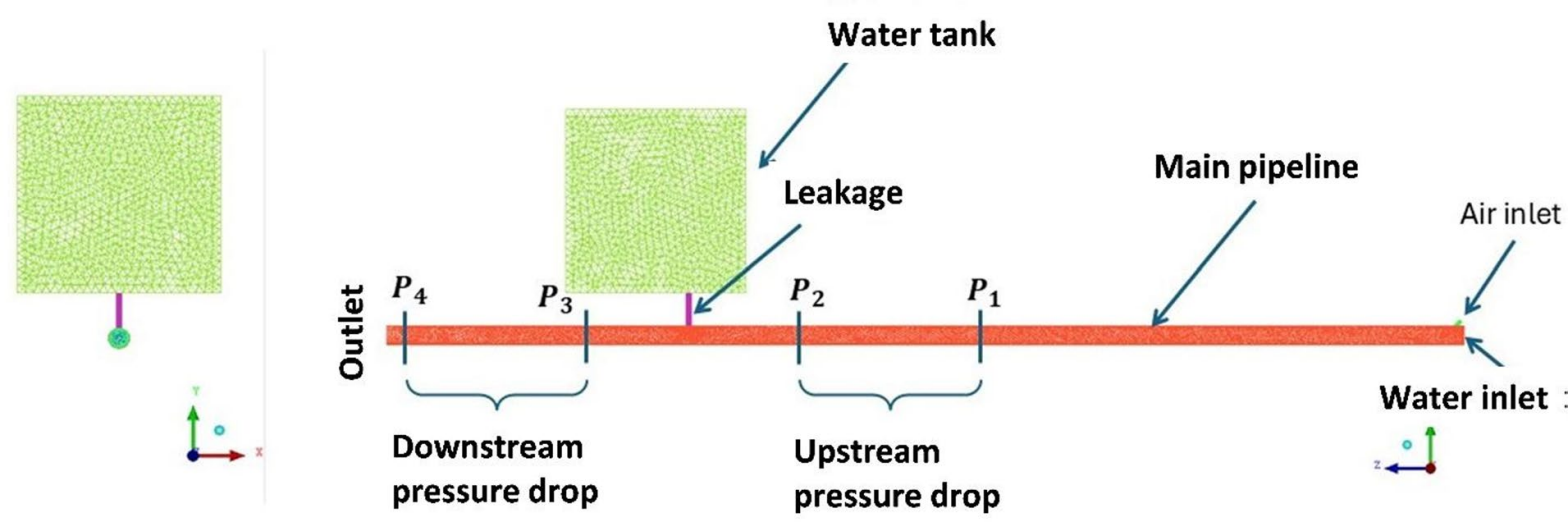
Mesh of the flow domain (single-leakage 15 mm).

To ensure a stable iterative calculation process and generate accurate numerical results, it is necessary to construct a mesh with high quality. Thus, tetrahedral elements with various mesh features were considered, including refined mesh, mesh with inflation layers, mesh refinement of the discharge area, and decreasing the transition ratio to capture high gradients of the outputs (Fig. 3). Considering these mesh features, seven meshes were generated to perform mesh sensitivity, as can be observed in Table 1.

**Fig. 4:**
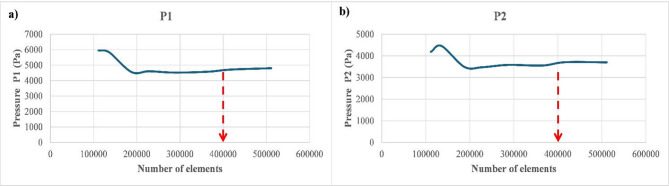
Mesh independency ($\:{V}_{SG}$=0.4 m/s and $\:{V}_{SG}$=3 m/s). (**a**) Pressure sensor 1. (**b**) Pressure sensor 2.

Figure 4a,b outline mesh sensitivity analysis considering pressure evolution in the locations P1 and P2 situated in the upstream region (1.55 m and 1.95 m from the inlet, respectively). As can be seen, a number of 4 × 10^5^ elements can be considered as an optimum number to ensure the independency of outputs from the selected mesh as well as to optimize computational time. In the current study, mesh 7 is considered for numerical simulations since it includes all mesh features and contains a number of elements above the optimum value.

**Table 1:** Mesh features with the equivalent number of elements.

Mesh	Mesh features							Equivalent number of elements
	Coarse mesh	Refined mesh	Inflation layers	Refinement of the discharge area	Transition ratio (1.12)	Transition ratio (1.11)	Transition ratio (1.1)	
Mesh 1	✖							135,799
Mesh 2	✖	✖						187,654
Mesh 3	✖	✖	✖					228,468
Mesh 4	✖	✖	✖	✖				286,935
Mesh 5	✖	✖	✖	✖	✖			364,895
Mesh 6	✖	✖	✖	✖	✖	✖		416,868
Mesh 7	✖	✖	✖	✖	✖	✖	✖	511,167

### Boundary conditions and solution procedure

The flow domain is modeled to be consistent with the experimental setup where separate intakes for gas and liquid phases are considered with a mass flow rate at the inlet boundary condition. Additionally, a pressure outflow is selected for the outlet boundary condition, while the no-slip conditions are adopted for the flow domain walls. Alternatively, the flow domain is considered to be full of water at the first stage, after a predetermined period of time, air is then injected into the flow domain via the air intake.

The Volume Of Fluid (VOF) approach is employed to model multiphase plug flow, taking into account discharge in the middle region of the pipeline. Moreover, the algorithm of pressure-velocity coupling: Semi-Implicit Method for Pressure Linked Equations (SIMPLE) is employed for determining outputs through a pressure-based algorithm. On the other side, the estimated Reynolds number is evaluated to be within the range of 52,032 to 156,814 based on the relationship of Shannak^38^, which is defined as the ratio of the sum of inertial forces of the two phases over the sum of viscous forces of the phases, as follows:

1 $$\:{Re}_{M}=\frac{\sum\:{F}_{I}}{\sum\:{F}_{V}}=\frac{{\rho\:}_{L}{{V}_{SL}}^{2}{{D}_{P}}^{2}+{\rho\:}_{G}{{V}_{SG}}^{2}{{D}_{P}}^{2}}{{\mu\:}_{L}{V}_{SL}{D}_{P}+{\mu\:}_{G}{V}_{SG}{D}_{P}}$$

where $\:{\rho\:}_{G}$ and $\:{\rho\:}_{L}$ represent the density of gas and liquid phases, respectively, $\:{V}_{SG}$ and $\:{V}_{SL}$ are the superficial velocity of gas and liquid phases, respectively, $\:{\mu\:}_{G}$ and $\:{\mu\:}_{L}$ stand for the viscosity of liquid and gas phases, respectively, and $\:D$ stands for the pipe diameter.

Thus, the $\:k-\omega\:$ SST turbulence model is considered since it can provide better results compared to other models, maintaining an optimum duration of the flow time^33^. Moreover, the present study examines different gas and liquid superficial velocities while accounting for various discharge scenarios, as can be seen in Table 2. A time step of 0.0001 s is implemented for all simulations, as it enables the attainment of a convergence criterion of 0.0001 for all residuals, which can be considered an acceptable stopping criterion for such type of numerical modeling^39^. Additionally, each scenario is executed for a total of 15 s to guarantee that a developed flow is achieved. Moreover, a super-calculator with 24 cores and parallel computation (Linux operating system with 64 GB of RAM and Intel Xeon E5 2690 V3 processor) was used to execute all cases, with each run requiring 24 to 96 h to meet stopping criterion requirements. Additional details of the numerical modeling and fluid properties are highlighted in Table 3.

**Table 2:** Cases of performed numerical simulations.

$\:{V}_{SG}\:(m/s)$	$\:{V}_{SL}\:(m/s)$	$\:{V}_{SG}(m/s)$	$\:{V}_{SL}\:(m/s)$	$\:{V}_{SG}\:(m/s)$	$\:{V}_{SL}\:(m/s)$
0.1	1	0.15	1	0.2	1
	1.5		1.5		1.5
	2		2		2
	3		3		3
0.3	1	0.4	1		
	1.5		1.5		
	2		2		
	3		3		

**Table 3:** Details of numerical simulations.

Parameter	Option	details
Solver	Pressure-based—Transient time scheme	Time step: 0.0001
Multiphase model	Volume Of Fluid (VOF)—Sharp Interface Modeling	Implicit body force
Phase-interaction	Surface Tension Force Modeling (CSF)	Constant surface tension coefficient = 0.073 N∕m
Turbulence model	$\:k-\omega\:$ model	SST
Boundary conditions	Inlet: imposed mass flow rate Outlet: Outflow Walls: Non-slip conditions	Constant values
Material properties	Gas phase: air Liquid phase: water	$\:\mathrm{A}\mathrm{i}\mathrm{r}:\:{\rho\:}_{G}=1.2\:kg/{m}^{3},\:{\mu\:}_{G}=1.7894{\times\:10}^{-5}kg/\left(m\bullet\:s\right)$ $\:\mathrm{W}\mathrm{a}\mathrm{t}\mathrm{e}\mathrm{r}:\:{\rho\:}_{L}=998.2\:kg/{m}^{3},\:{\mu\:}_{L}=0.001\:kg/\left(m\bullet\:s\right)$
Solution methodology	Coupling of pressure-velocity: PISO Pressure spatial discretization: PRESTO! Volume fraction spatial discretization: Geo-reconstruct Gradients: Least-squares cell-based Other variables: first and second-order upwind	Convergence criterion: residuals (0.0001)

### Experimental setup

**Fig. 5:**
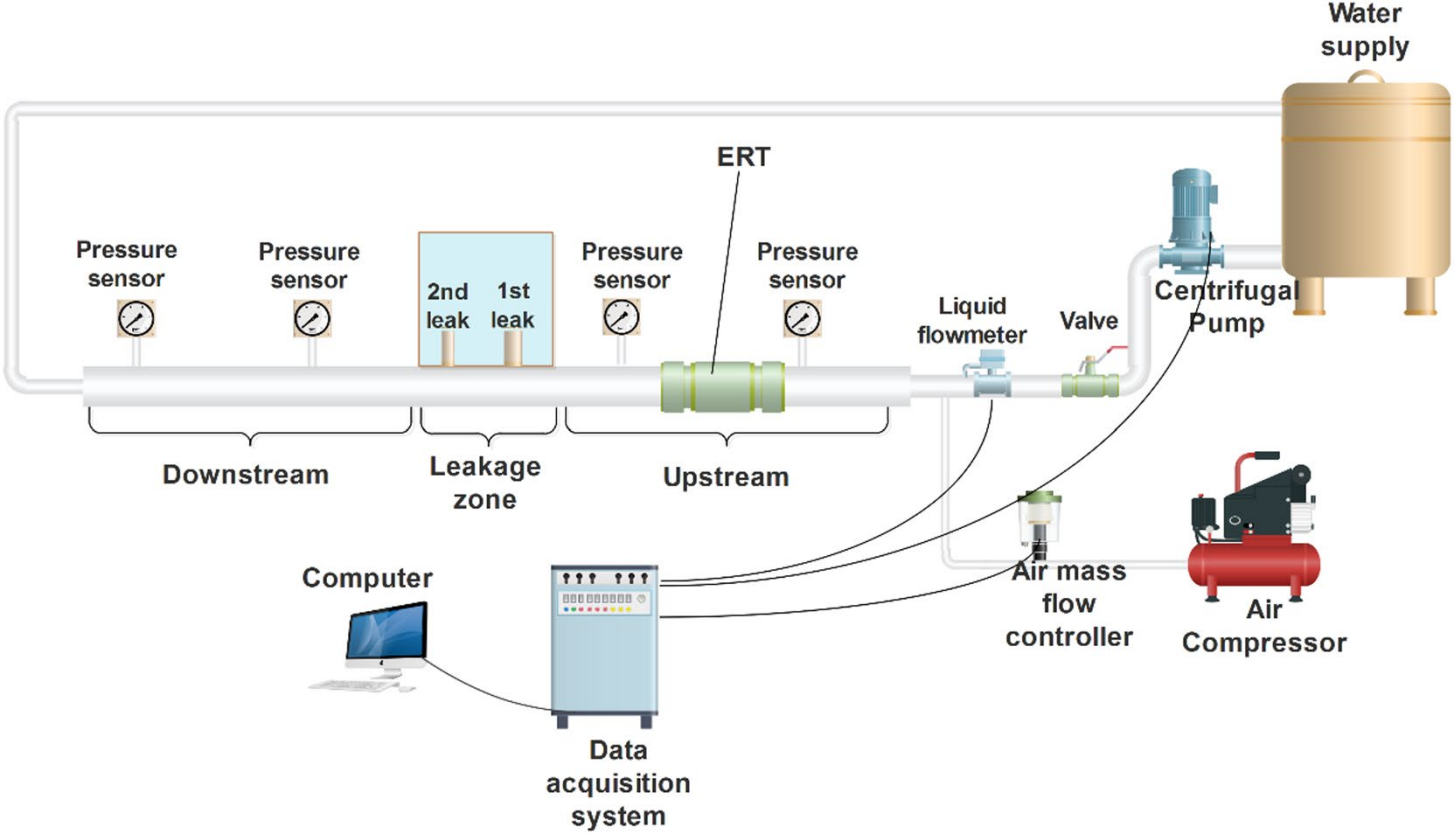
Schematic representation of the flow loop system.

A series of tests were conducted to verify the generated computational findings employing an experimental flow loop system in the research Lab of advanced multiphase flow and flow assurance laboratory at Hamad Bin Khalifa University. This setup contains two major parts; the first consists of the main pipeline (50.8 mm in diameter and 6 m in length) with artificial leakages that are controlled remotely, and the second is the water tank (has geometrical characteristics of 0.5 × 0.5 × 1 m) that is installed on the discharge area to visualize the flow coming from the leakages to mimic underwater conditions. Additionally, this flow loop system is associated with different types of sensors that can measure dynamic pressure, mass flow rate, temperature, and gas volume fraction. Moreover, a DSLR and high-speed cameras were used to record the flow within the main pipeline and the water tank, respectively. The dynamic pressure sensors associated with experimental setup are installed in the upstream (before leakage) and downstream (after leakage) regions to characterize the flow before and after leakage area. These sensors can operate and record pressure fluctuations within a range of 0–16 bars with an error of 0.15% of span BFSL (best fit straight line). Figure 5 highlights a schematic representation of the experimental setup.

### Experiments procedure

In the first stage, the liquid tank was filled with water up to 250 L, after that, the liquid phase (water) is injected into the flow loop system via a centrifugal pump at different flow rates starting from 134 to 323.5 kg/min. Before starting experiments, the ERT system is calibrated with one phase (water as a primary phase) to set up a reference of the gas void fraction^40^. After that, the gas phase is introduced via a mass flow rate controller in accordance with the needs of the experiments, where the amount of the gas phase (air) can be adjusted from 10 to 50 g/min. Once the multiphase flow is established in the main pipeline, readings from various measuring devices are collected, and the recording of cameras is started. After that, the discharge associated with the pipeline is opened for a duration of 30 s. Once the discharge valve is closed, the recording of data is ended for all devices, and the injection of liquid and gas phases is stopped.

### Validation

**Fig. 6:**
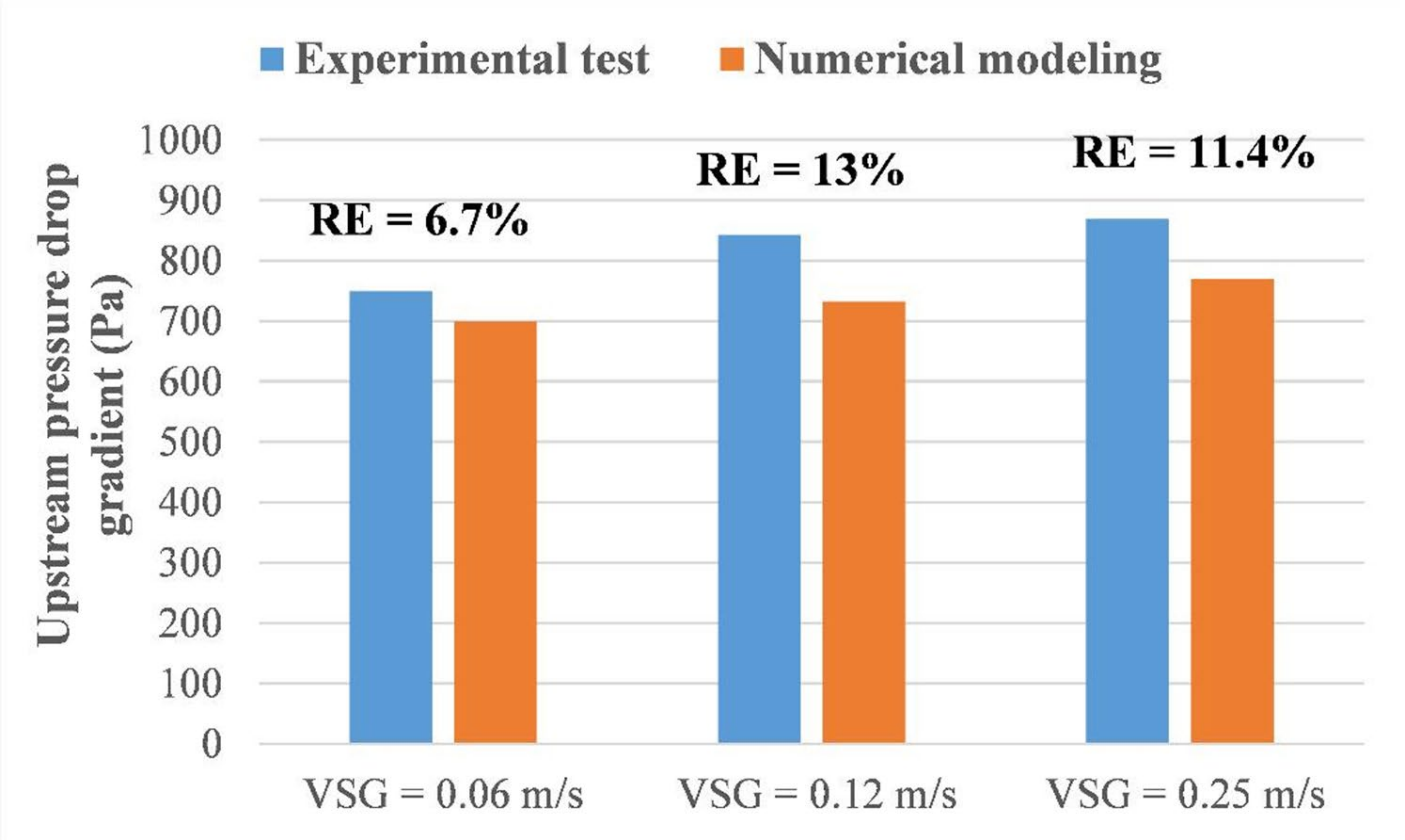
Validation of the obtained numerical results with experimental data considering pressure drop in the upstream section (V_SL_ = 1.94 m/s) for the case of single leakage of 3 mm.

Before proceeding with numerical simulations, validation of the numerical model is performed against a set of experimental data collected from a sophisticated experimental flow loop system that contains sensitive sensors. Firstly, the determined pressure drop from the numerical model is compared with experimental data of a pressure drop transducer installed on the upstream section of the pipeline (the distance between the two sensors is 1.9 m), as shown in Fig. 6. In this Figure, the Relative Error (RE) is estimated based on the results from both approaches using the following expression:

2 $$\:RE=\left|\frac{{V}_{e}-{V}_{p}}{{V}_{e}}\right|\times\:100$$

where $\:{V}_{e}$ stands for the value of the experimental pressure drop reading and $\:{V}_{p}$ is the predicted value.

It is determined that the mean relative error of the considered cases is found to be 10%, which outlines a satisfactory concordance between the experimental data and the numerical model. This proves the ability of the latter to accurately forecast the pressure variation of the liquid-gas system within the considered fluid domain.

**Fig. 7:**
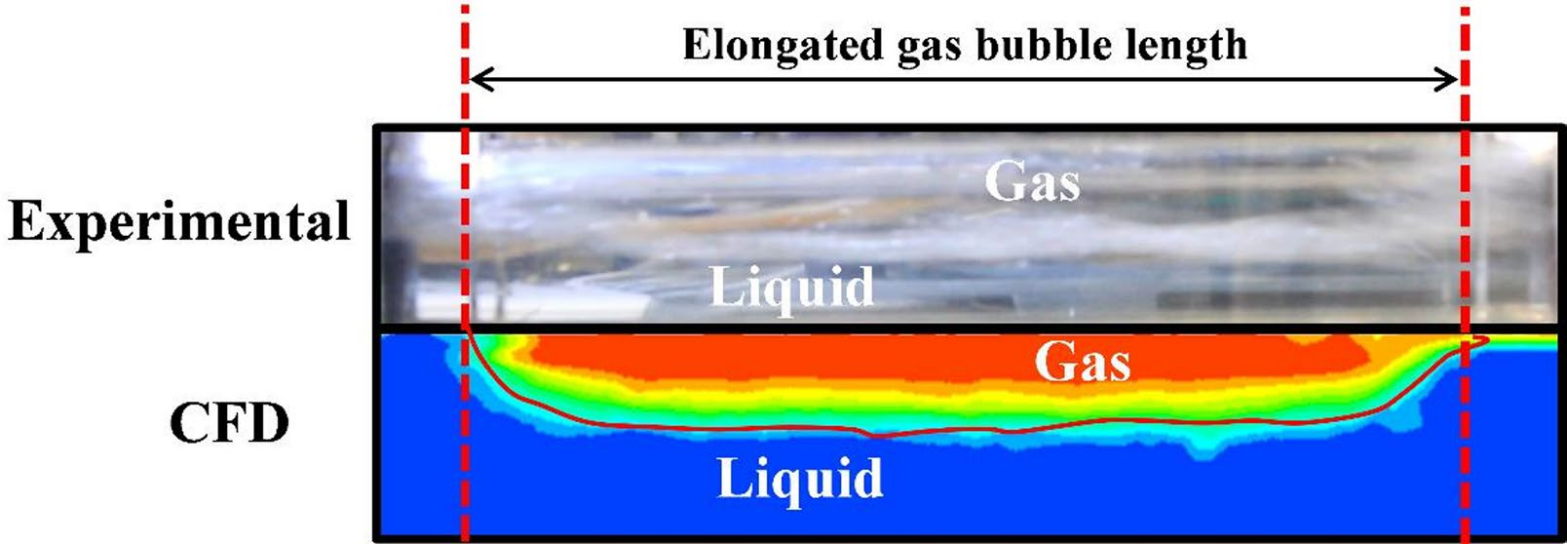
Comparison of the flow configuration in the main pipeline for both numerical and experimental methods (V_SG_ = 0.3 m/s, V_SL_ = 1.5 m/s).

Additionally, the flow configuration in the main pipeline (middle plane of the 3D flow domain) is compared with the flow configuration in the downstream section of the pipeline (section situated after the discharge area) obtained via a DSLR camera, as can be observed in Fig. 7. In this Figure, the length of the elongated bubble matches the experimental value, which is estimated to be around 0.26 m. Moreover, additional comparisons with experimental data for the gas volume fraction in the main pipeline and the gas release velocity in the water tank are performed to validate the developed nomograph (the last section of the paper).

**Fig. 8:**
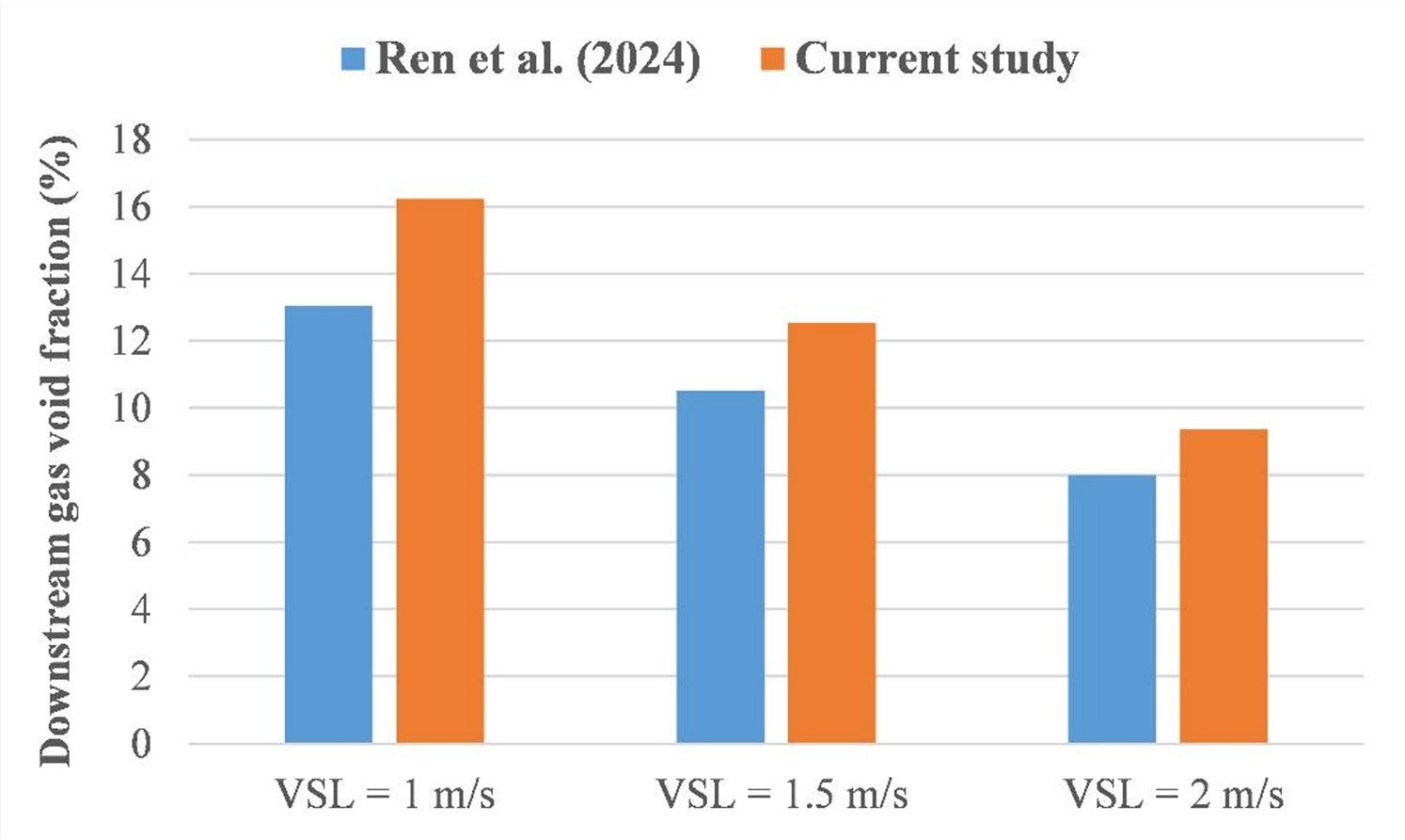
Comparison of the gas void fraction in the downstream region for the current analysis with the study of Ren et al.^34^ (V_SG_ = 0.3 m/s).

An additional comparison with the numerical work of Ren et al.^34^ is performed for the gas void fraction in the downstream region (Fig. 8). It can be observed that the obtained results concord with each other and match the behavior of the gas void fraction as the V_SL_ increases with a certain discrepancy. The latter can be attributed mainly to the difference in pipeline diameter, which is considered to be 50.8 mm and 50 mm for the current analysis and the study of Ren et al.^34^, respectively.

## Results and discussion

**Fig. 9:**
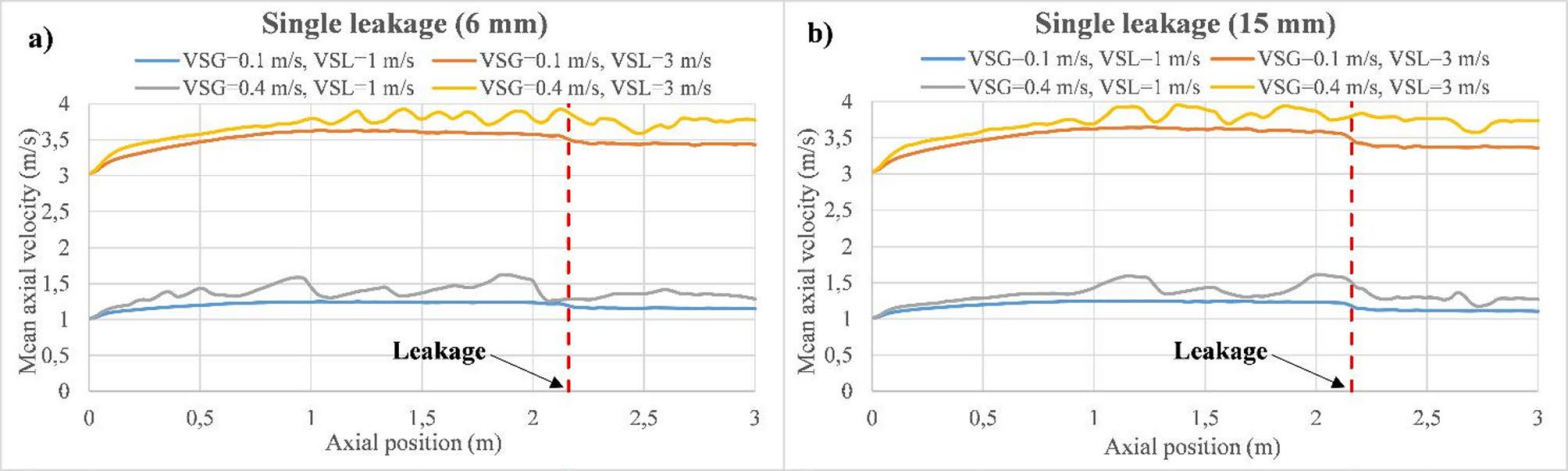
Evolution of the mixture axial velocity through the main pipeline for discharge cases of 6 and 15 mm. (**a**) Case of 6 mm. (**b**) Case of 15 mm.

The mean axial velocity of the mixture along the pipeline is exhibited in Fig. 9 for various gas and liquid superficial velocities considering two cases of discharge, including single-leakage of 6 mm (Fig. 9a) and single-leakage of 15 mm (Fig. 9b). As can be observed, the mean axial velocity of the mixture is dominated by the $\:{V}_{SL}$, while the $\:{V}_{SG}$ has a slight effect due to the fact that plug flow is characterized by elongated gas bubbles moving within the liquid phase. Moreover, the mixture reaches a stable axial velocity after the axial position of 1 m, where the cases with the highest gas superficial velocity ($\:{V}_{SG}$ = 0.4 m/s) start to fluctuate because they are close to the transition phase to the slug flow regime^41^. Once the mixture reaches the discharge location, the axial velocity is slightly influenced, particularly for the discharge case of 6 mm, where a high fluctuation frequency characterizes the latter compared to the case of the discharge of 15 mm.

### Transient behavior

The present analysis is based on raw dynamic pressure time-series signals recorded in various places within the pipeline. These raw signals inherently capture the transient hydrodynamic behavior of two-phase plug flow, including pressure oscillations induced by elongated gas bubbles, liquid slugs, and leakage-related mass loss. Rather than relying on direct pressure level changes alone, which may be masked by natural flow fluctuations, several features were extracted to enhance leakage detectability.

**Fig. 10:**
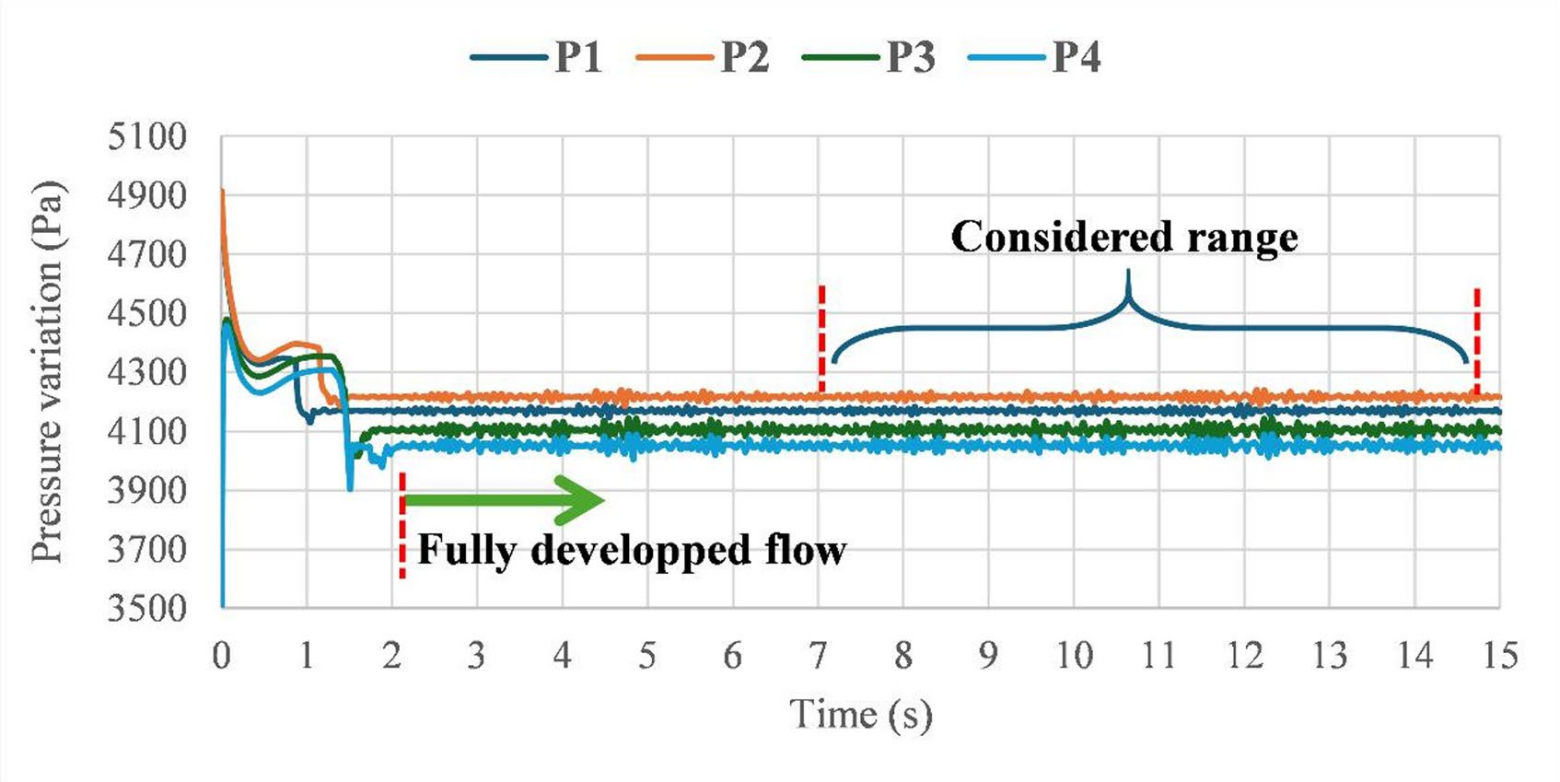
Pressure variation during flow time in positions P_1_, P_2_, P_3_, and P_4_.

Pressure variation during a flow time of 15 s is recorded in positions P_1_, P_2_, P_3_, and P_4_, as can be seen in Fig. 10. As can be observed, the multiphase flow takes around 2 s to be fully developed in the main pipeline from its entrance. On the other side, the last 8 s of the flow time are considered for the investigation to ensure that the flow is under fully developed conditions.

**Fig. 11:**
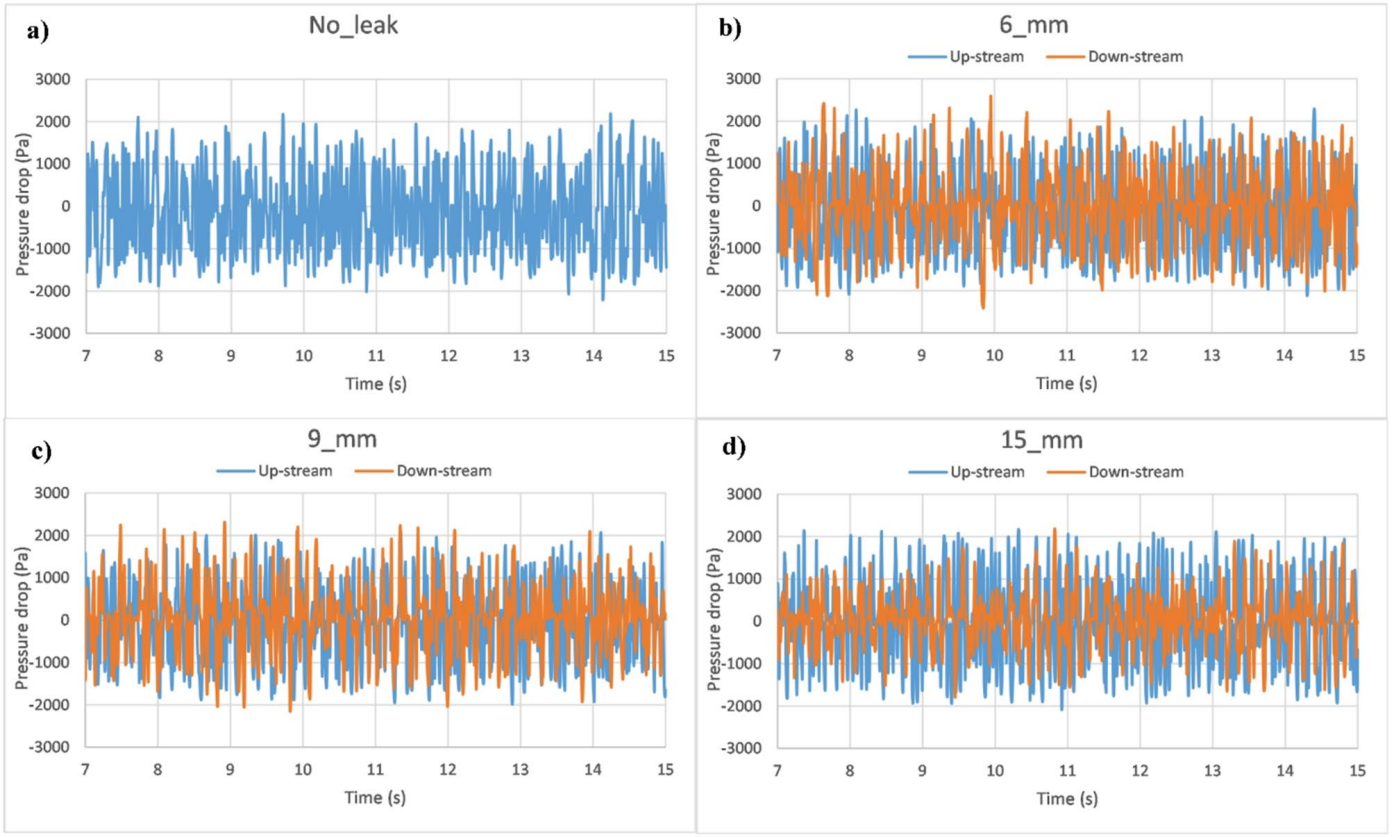
Pressure drop variation in both upstream and downstream regions for various discharge cases ($\:{V}_{SG}=0.4\:m/s$ and $\:{V}_{SL}=3\:m/s$). (**a**) case of no-leak. (**b**) case of single-leakage of 6 mm. (**c**) case of single-leakage of 9 mm. (**d**) case of single-leakage of 15 mm.

For instance, Fig. 11a–d show the determined pressure drop signals for the case of $\:{V}_{SG}=0.4\:m/s$ and $\:{V}_{SL}=3\:m/s$, where it fluctuates within the range of (− 2500 to 2500 Pa). It can be observed that pressure drop signals are slightly influenced by discharge occurrence, contrary to the downstream region, where it is found to decrease with discharge size. Additionally, it is a challenging task to characterize a discharge occurence based only on pressure variation, and consideration of additional analyzing approaches is needed to enhance the accuracy of prediction and classification.

### Gas void fraction behavior

**Fig. 12:**
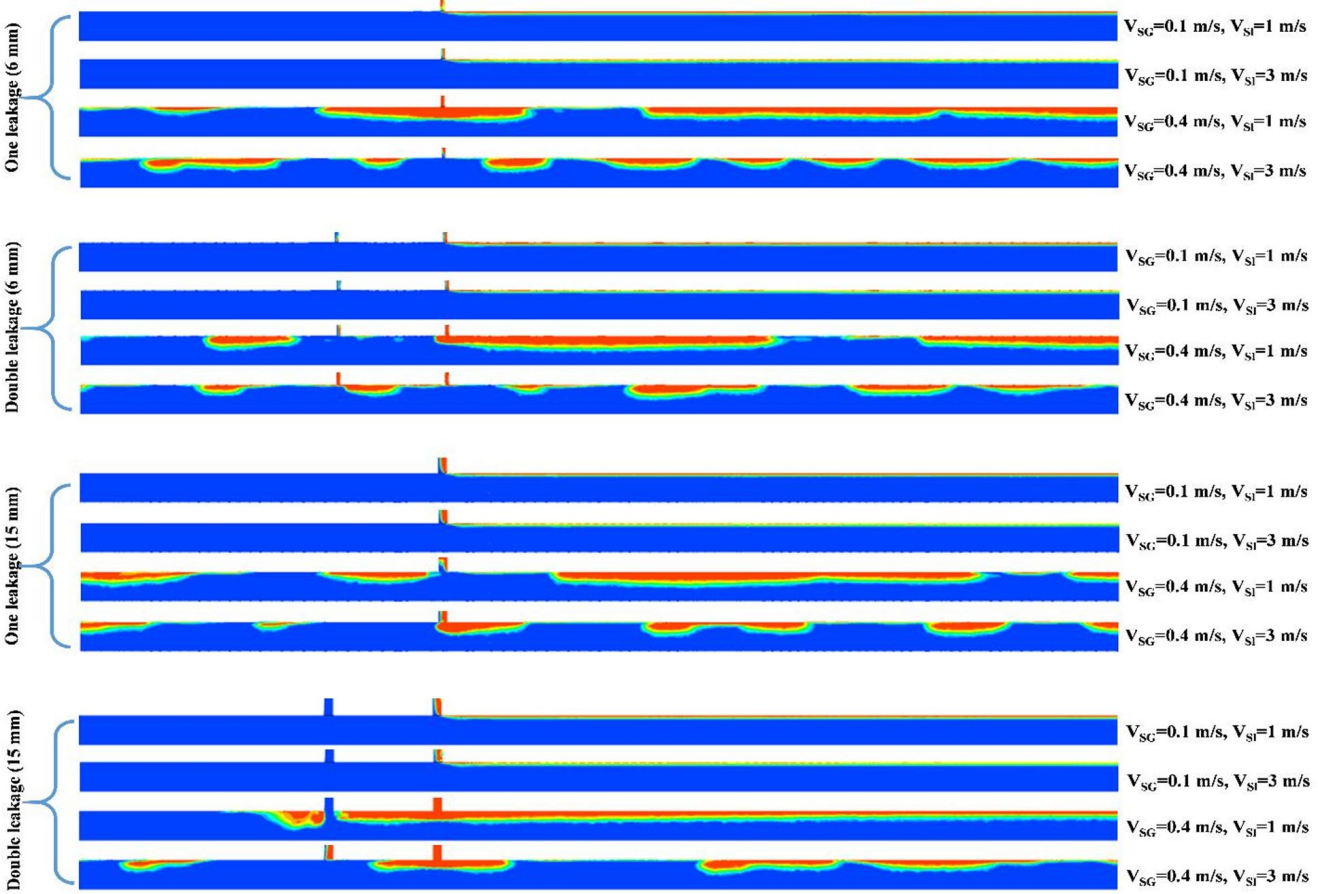
Behavior of the flow structure in the main pipeline for various scenarios of discharge (single and double leakages of 6 mm and 15 mm in diameter) considering 4 extreme cases of the plug flow regime.

Figure 12 exhibits the gas void fraction evolution in the main pipeline for various scenarios of discharge (single and double leakages of 6 mm and 15 mm in size) considering 4 extreme cases of the plug flow regime. It can be observed that the cases with a V_SG_ of 0.1 m/s (the lowest value of the V_SG_), most of the gas phase amount escapes through the discharge area and the liquid phase occupies the downstream section. Moreover, in the case of double-leakage, a slight amount of the gas phase is released via the second leakage of 6 mm; however, the entire amount of the gas phase discharges through the first leakage of 15 mm. Once the V_SG_ is increased to 0.4 m/s while the V_SG_ is kept constant at 1 m/s, a full plug morphology appears with a high value of the elongated bubble length. At the flow conditions of V_SG_ = 0.4 m/s, V_SL_ = 3 m/s, the elongated bubble length decreases while length of liquid slug increments with the size and number of leakages.

**Fig. 13:**
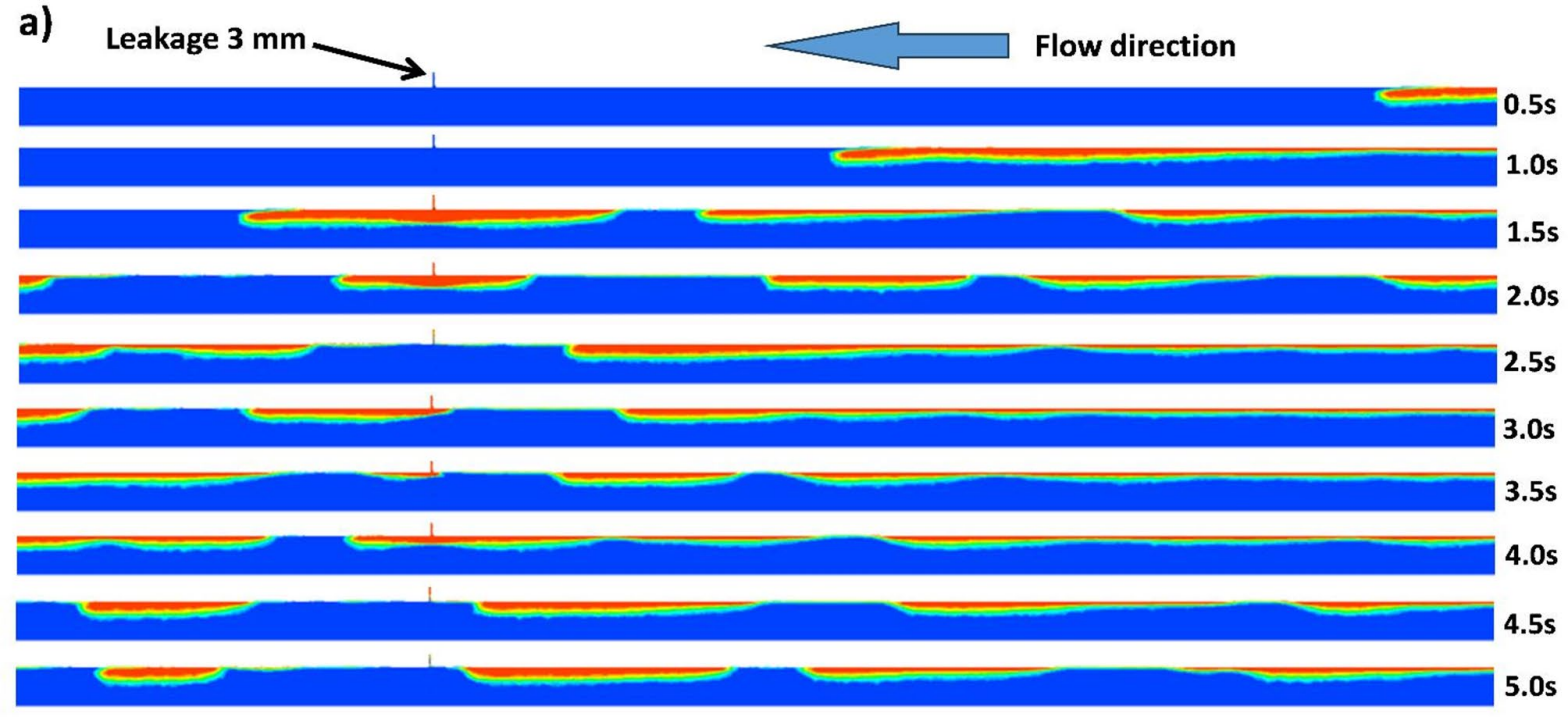

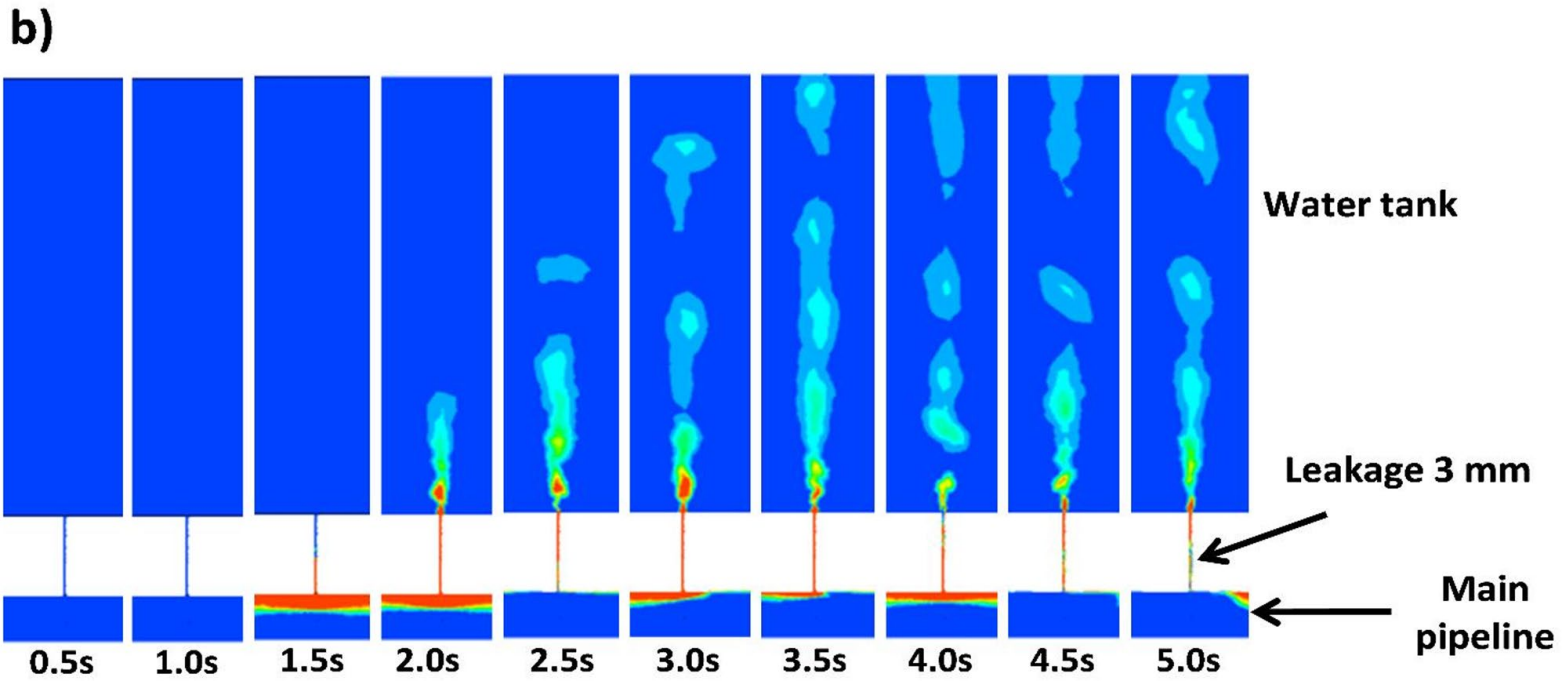
(**a**) Development of the flow configuration during time for the main pipeline (V_SG_ = 0.3 m/s, V_SL_ = 1.5 m/s). (**b**) Development of the flow configuration during time for the water tank (V_SG_ = 0.3 m/s, V_SL_ = 1.5 m/s).

Initially, the flow configuration within the fluid domain is visualized to ensure that the plug flow regime is attained. For that, the middle plane is considered for visualization of gas void fraction contours, taking into account both the main pipeline and water tank, as shown in Fig. 13a,b. A closer look at Fig. 13a indicates that the flow configuration attains a stable plug morphology after a certain time of 2.5 s, which is defined by a single unit containing a gas-elongated bubble followed by a liquid slug^16,42^. Figure 13b outlines the dynamics of the gas phase in the water tank over time for the case of V_SG_ = 0.3 m/s, V_SL_ = 1.5 m/s. In this scenario, the gas phase takes around 1.5 s to reach the discharge position from the inlet (2.16 m) in the main pipeline. After that, a portion of the gas phase enters through the leakage, and it takes another 2 s to reach the top of the water tank (0.62 m from the centerline of the pipeline). The analysis of the gas phase dispersion (gas phase release) in the water tank is analyzed in the following sections.

To investigate the effect of a leaky pipeline carrying multiphase flow more deeply, the gas phase void fraction is determined in both upstream and downstream regions of the main pipeline. To consider the transient nature of the plug flow, the gas void fraction is estimated to be over 8 s of the time flow (from 7 s to 15 s) using the flowing formula:

3 $$\:\alpha\:=\frac{1}{V}{\int\:}_{7}^{15}{V}_{i}.dt$$

where $\:\alpha\:$ stands for the void fraction of the gas phase, $\:V$ is the pipeline’s total volume, $\:{V}_{i}$ represents the equivalent gas phase volume at the instant (t).

Moreover, the discharge effect is evaluated in terms of the percentage of gas void fraction decrease from the upstream to the downstream region ($\:\beta\:$), as follows:

4 $$\:\beta\:=\frac{{\alpha\:}_{Up}-{\alpha\:}_{Down}}{{\alpha\:}_{Up}}\times\:100$$

where $\:{\alpha\:}_{Up}$ and $\:{\alpha\:}_{Down}$ represent the gas volume fraction in the upstream and downstream regions, respectively.

**Fig. 14:**
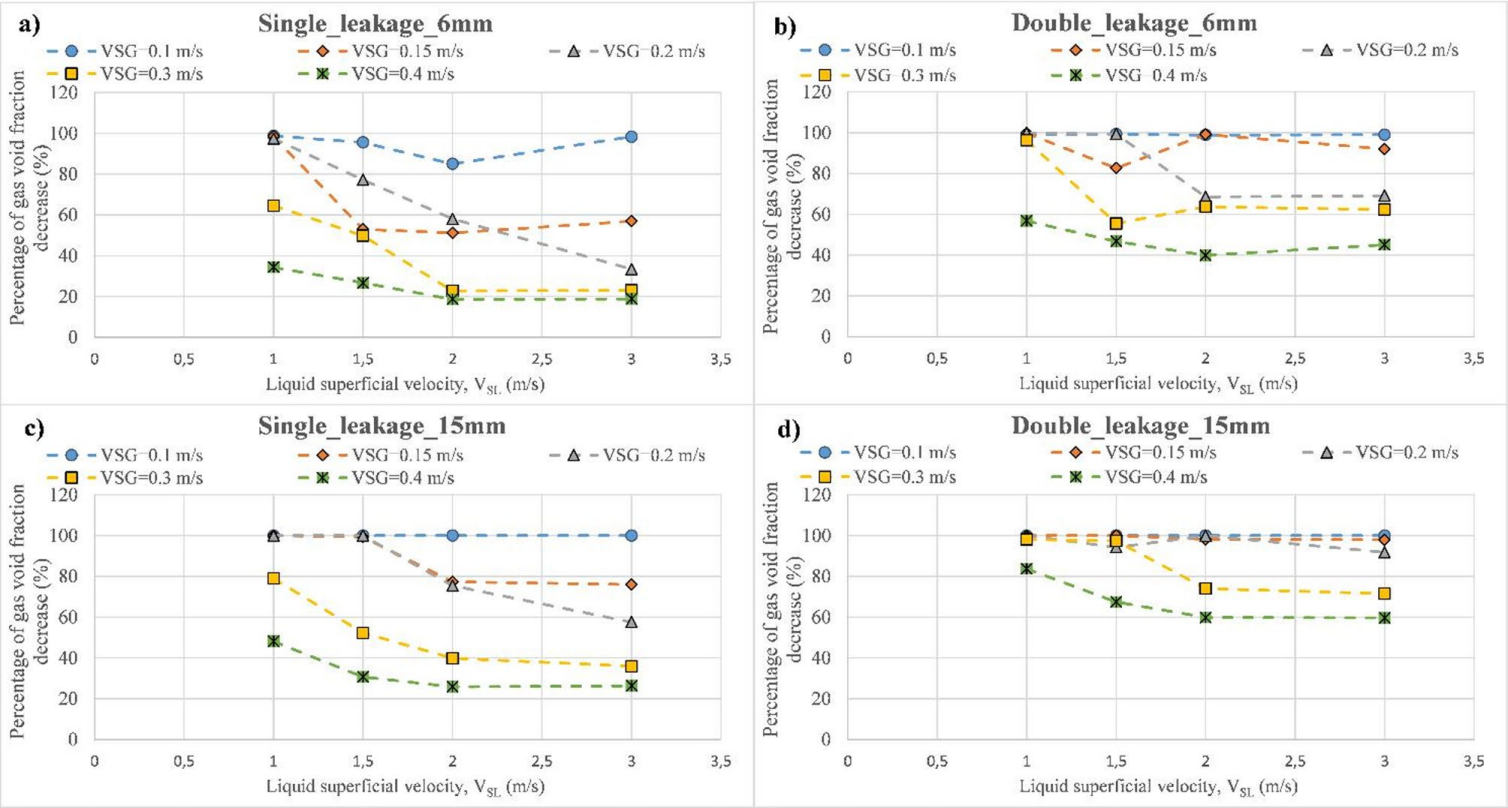
Behavior of the percentage of gas void fraction decrease as a function of the liquid superficial velocity for various discharge scenarios. (**a**) single-leakage of 6 mm. (**b**) double-leakage of 6 mm. (**c**) single-leakage of 15 mm. (**d**) double-leakage of 15 mm.

For instance, $\:\beta\:=100\%$ means that the gas phase is released through the discharge region. Figure 14 outlines the percentage of gas void fraction decrease ($\:\beta\:$) for various gas and liquid superficial velocities considering different discharge scenarios, including single-discharge of 6 mm, single- discharge of 15 mm, double-discharge of 6 mm, and double-discharge of 15 mm. As can be seen, the lowest value of the $\:{V}_{SG}$ shows the highest percentage of gas void fraction decrease for all the discharge scenarios, indicating that all the gas amount in the main pipeline is released through the discharge region, particularly for the cases of single-discharge of 15 mm, double-discharge of 6 mm, and double-discharge of 15 mm. However, with the increase of the $\:{V}_{Sg}$, the percentage of gas void fraction decrease ($\:\beta\:$) diminishes, particularly for high values of the $\:{V}_{SL}$ where this effect is more prominent in the case of a single-discharge of 6 mm. This behavior can be explained by the fact that once the flow regime map is close to the bubbly flow (high values of liquid and gas superficial velocities), most of the gas amount is carried within the main pipeline^21^. This indicates that in the case of a leaky pipeline carrying multiphase flow, higher flowrates gas and liquid phases are required to reduce gas release through discharge leaks. On the other hand, it is noticed that the case of a double-discharge of 6 mm shows a higher gas release compared to the case of a single-discharge of 15 mm.

### Standard deviation of pressure signals

**Fig. 15:**
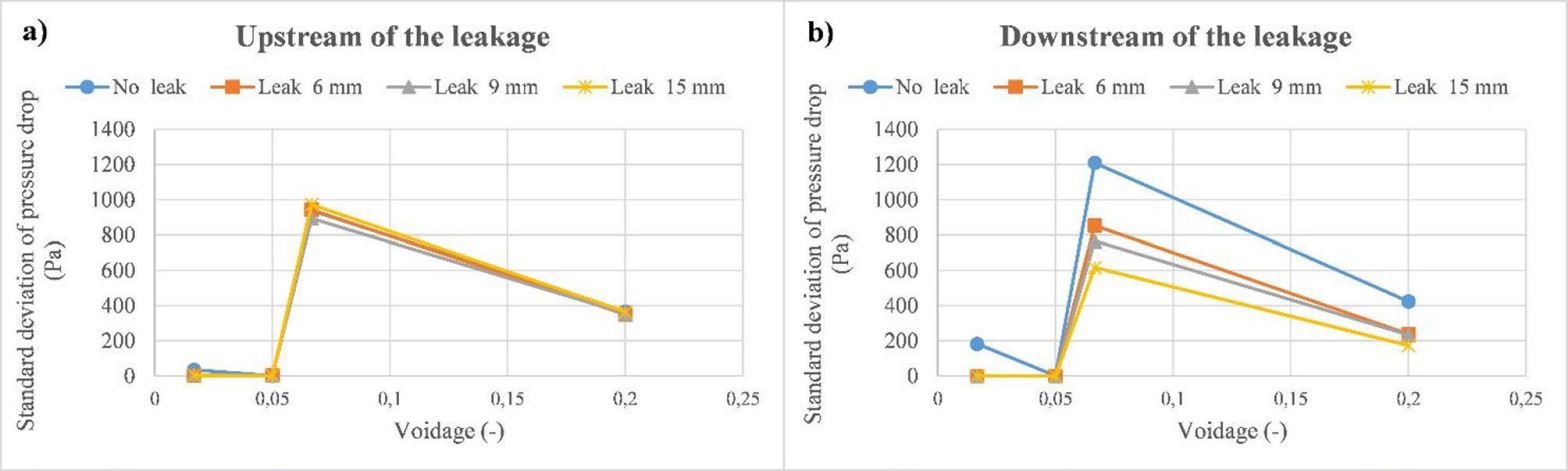
Standard deviation of pressure drop as a function of voidage for various discharge cases. (a) upstream section. (**b**) downstream section.

As discussed previously, the upstream section of the pipeline is less sensitive to the occurrence of discharge as compared to the downstream section. Moreover, this effect is also observed when determining the standard deviation of pressure drop signals, as seen in Fig. 15a,b. For that, the downstream part is considered for the analysis in the coming sections using different methods.

**Fig. 16:**
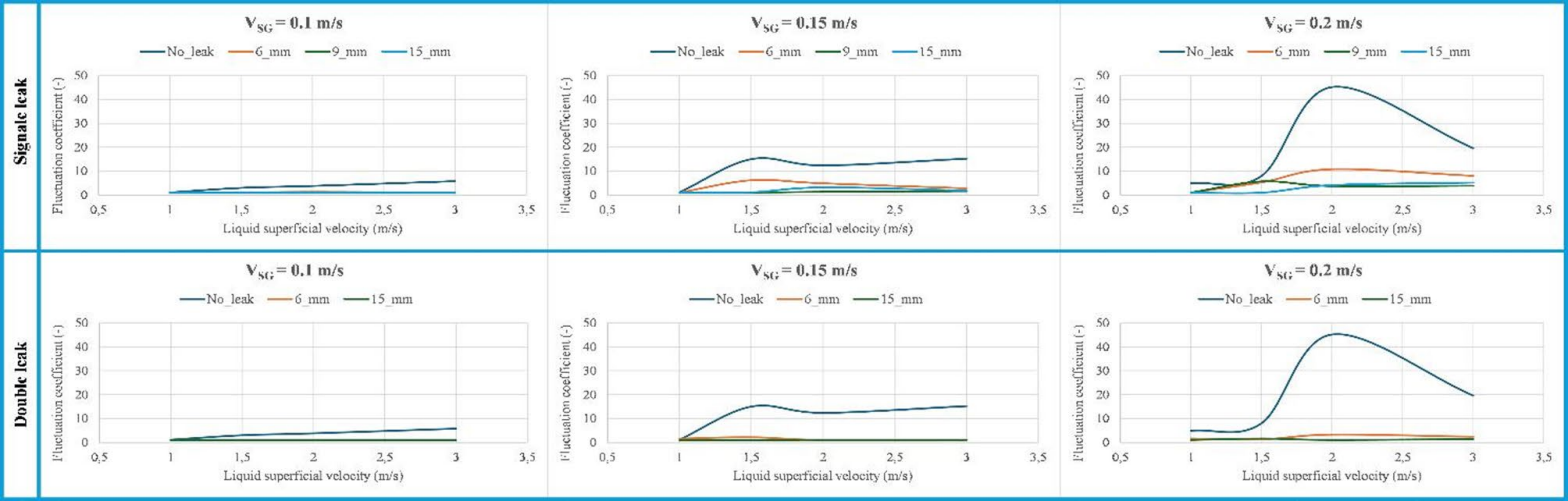
Fluctuation coefficient as a function of liquid superficial velocity for various discharge cases.

To characterize the impact of liquid and gas superficial velocities on the oscillation of pressure drop signals of multiphase flows in horizontal pipelines, a dimensionless fluctuation coefficient ($\:\varPsi\:$) is considered for various cases of discharge including no-leak, 6 mm, 9 mm, and 15 mm (Fig. 16). The fluctuation coefficient ($\:\varPsi\:$) is considered to be:

5 $$\:\varPsi\:=\frac{RMS}{A}$$

where $\:RMS=\sqrt{\frac{1}{n}{\sum\:}_{i}{x}_{i}^{2}}$ and $\:A=\frac{{\sum\:}_{i=1}^{n}abs\left({x}_{i}\right)}{n}\:$ are the root mean square and absolute mean value of the recorded signals, respectively.

Figure 16 points out that the fluctuation coefficient of the pressure drop signals intensifies with superficial velocities of liquid and gas in the no-leak case. However, this effect is attenuated by the existence of discharge, where this effect decreases with its size. This can be explained by the fact that the higher the discharge size, the more air is evacuated through the discharge area, particularly for plug flow, which escapes from the main pipeline once it reaches the discharge point. Moreover, it is observed that fluctuation is more sensitive to the gas superficial velocity.

### Continuous wavelet transform (CWT)

In addition, time–frequency features obtained using the continuous wavelet transform (CWT) were considered to capture the non-stationary nature of plug flow. Unlike classical Fourier-based methods, CWT enables simultaneous localization of dominant frequencies and their temporal evolution, which is particularly important in intermittent flows where leakage-induced disturbances may appear intermittently or overlap with slug-induced oscillations. The selected wavelet-based features therefore provide complementary information to time-domain statistics by highlighting changes in oscillation frequency bands and energy distribution associated with leakage size and operating conditions.

In this subsection, the CWT representation of the determined times series signals is taken into account due to the fact that it has the capability to visualize pressure time series signals into coefficients (magnitude) in both time and frequency domains (scalogram). Thus, a MATLAB code is considered to generate scalograms for various operating conditions of gas and liquid superficial velocities, taking into account different scenarios of discharge.

**Fig. 17:**
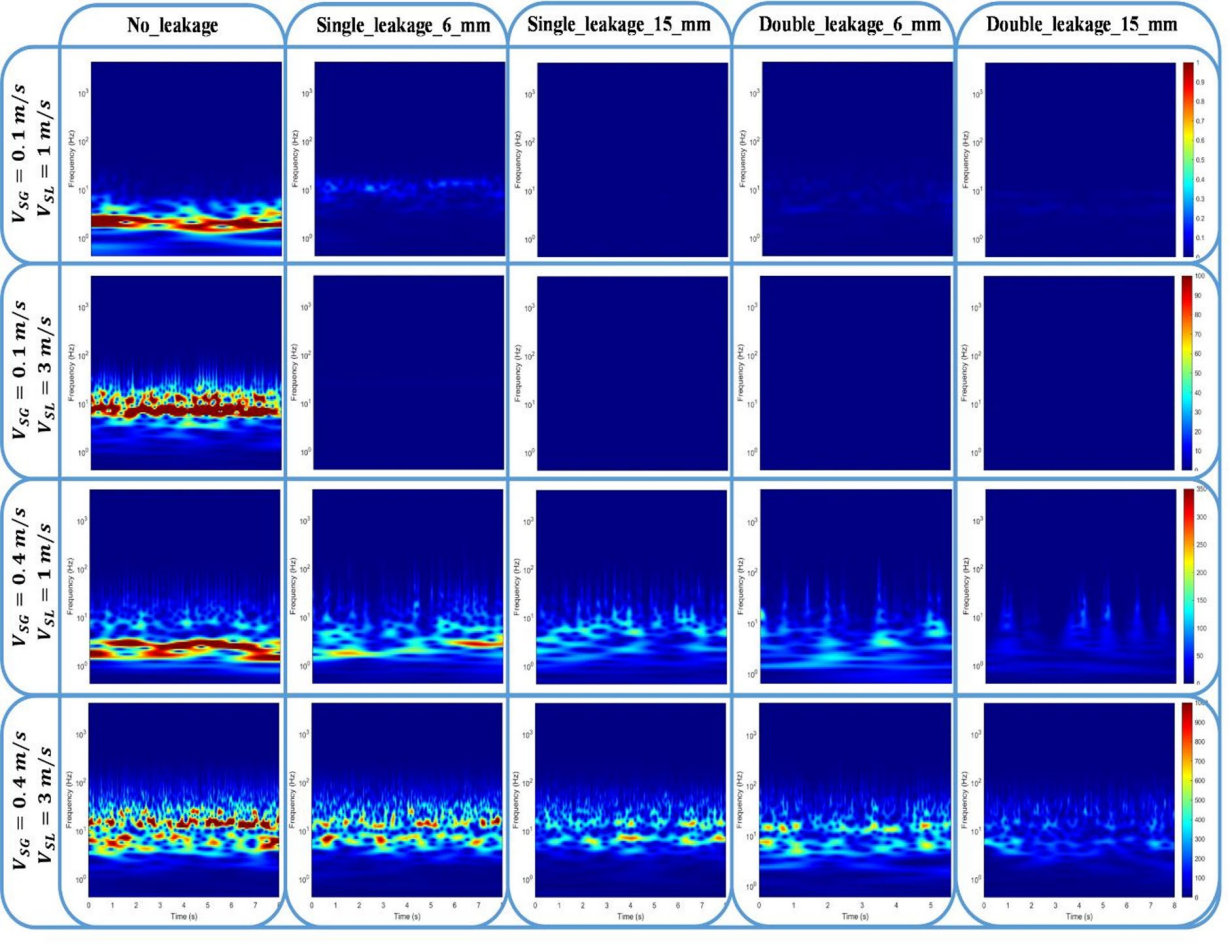
CWT-based scalograms of pressure drop signals for various discharge scenarios considering four operating conditions scenarios of superficial gas and liquid velocities.

Figure 17 shows the determined scalograms of 5 cases, including no-discharge, single-discharge (6 mm), single-discharge (15 mm), double-discharge (6 mm), and double-discharge (15 mm) where four operating condition scenarios of superficial gas and liquid velocities were considered. For comparison purposes, the same magnitude range is applied for each case. As can be seen, the no-discharge case shows higher magnitudes compared to other cases due to the release of the gas phase, resulting in less fluctuation within the pipeline. For instance, the CWT-based scalogram of the no-discharge case at $\:{V}_{SG}=0.1\:m/s$ and $\:{V}_{SL}=1\:m/s$ shows a smooth variation over time with a frequency of around 2.3 Hz. However, when the $\:{V}_{SL}$ increases to 3 m/s, the scalogram indicates an amplified oscillation with a higher frequency (around 10 Hz). This can be attributed to the additional amount of liquid phase in the pipeline, causing the pressure signal to fluctuate with higher intensity. On the other hand, the cases with discharge at the lowest superficial gas velocity ($\:{V}_{SG}=0.1\:m/s$) show almost a negligible variation compared to the case of no-discharge. In these cases, it can be a challenge to characterize the discharge using the CWT Method, particularly for ($\:{V}_{SG}=0.4\:m/s$) due to the low amount of gas phase flowing through the pipeline.

For the case of $\:{V}_{SG}=0.4\:m/s$ and $\:{V}_{SL}=1\:m/s$ the scalograms show a similar trend as the case of $\:{V}_{SG}=0.1\:m/s$ and $\:{V}_{SG}=0.1\:m/s$ with higher magnitudes (maximum value is found to be around 500) where the latter diminishes with discharge number and size. Furthermore, the case of $\:{V}_{SG}=0.4\:m/s$ and $\:{V}_{SL}=3\:m/s$ results in a wider frequency band of high magnitudes (approximately from 2 to 10 Hz) with additional fluctuations over time, however, this effect vanishes with discharge size and number. Subsequently, the frequency of the analyzed signals is mainly affected by the liquid superficial velocity and its range is slightly influenced by the discharge size and number.

### Probability density function (PDF)

Statistical features such as the standard deviation, root mean square (RMS), probability density function (PDF), and cumulative probability density function (CPDF) were selected because they quantify the dispersion, variability, and distribution of pressure fluctuations over time. These features are physically linked to changes in flow instability and phase redistribution caused by leakage. For example, an increase or attenuation in pressure fluctuation intensity reflects alterations in gas holdup and momentum exchange between phases, making statistical descriptors effective indicators of leakage occurrence.

The CPDF shows the percentage of the pressure drop that is less than a given value. In contrast, the PDF of the pressure drop is thought to be the likelihood that the pressure drop will fall within a given range. This approach is frequently used for gas-liquid multiphase systems for a number of parameters, such as air void percentage and pressure drop^43^.

**Fig. 18:**
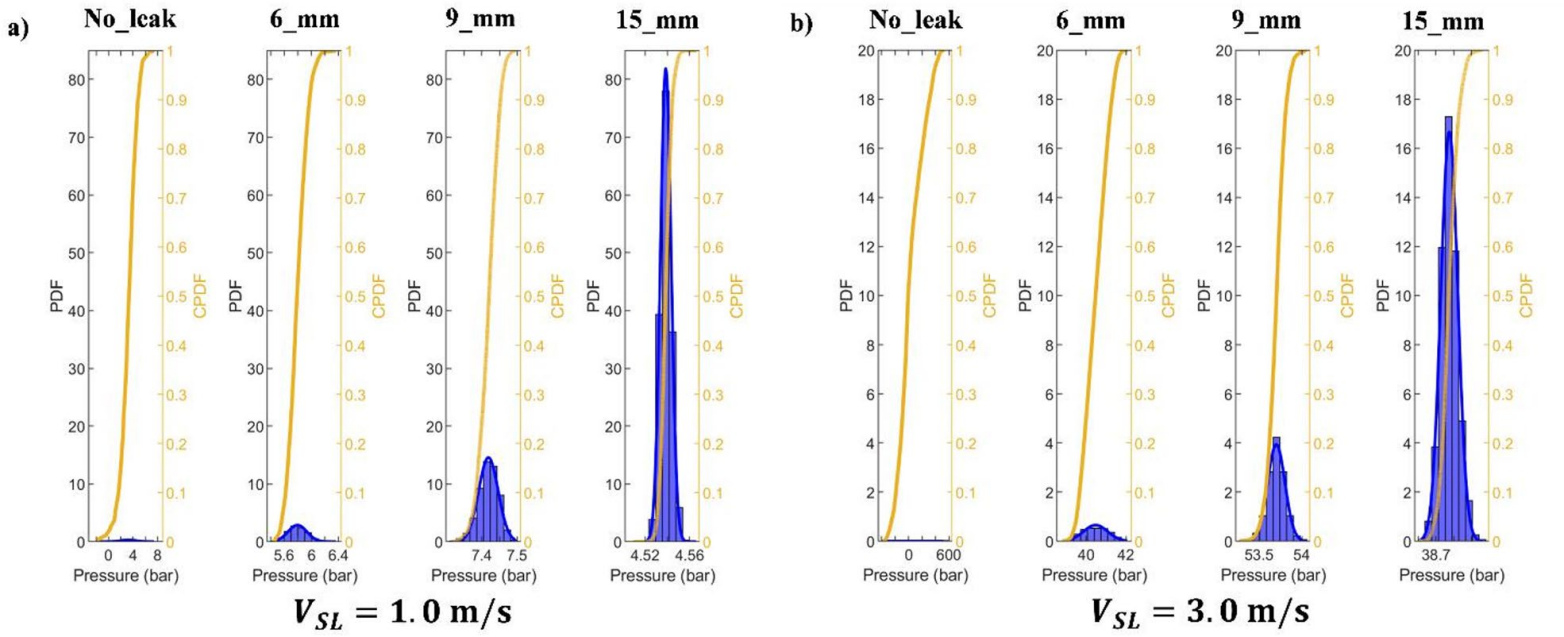
PDF and CPDF of the collected pressure fluctuation considering $\:{V}_{SG}$ of 0.1 m/s. (**a**) $\:{V}_{SL}=1\:m/s$. (**b**) $\:{V}_{SL}=3\:m/s$.

Figure 18a,b show the PDF and CPDF of the collected pressure fluctuation considering $\:{V}_{SG}$ of 0.1 m/s for the following leak scenarios: no-leak, 6 mm, 9 mm, and 15 mm. It can be observed that the PDF’s highest value increments with the discharge size where the case of $\:{V}_{SL}=1.0\:m/s$ shows higher levels as compared to the case of $\:{V}_{SL}=3.0\:m/s$. For example, the PDF’s highest value increases by around 2726% and 2449% when the discharge size increments from 6 mm to 15 mm, for the $\:{V}_{SL}=1.0\:m/s$ and $\:{V}_{SL}=3.0\:m/s$, respectively. Moreover, the no-leak case is characterized by a flat curve, which makes it easier to identify.

**Fig. 19:**
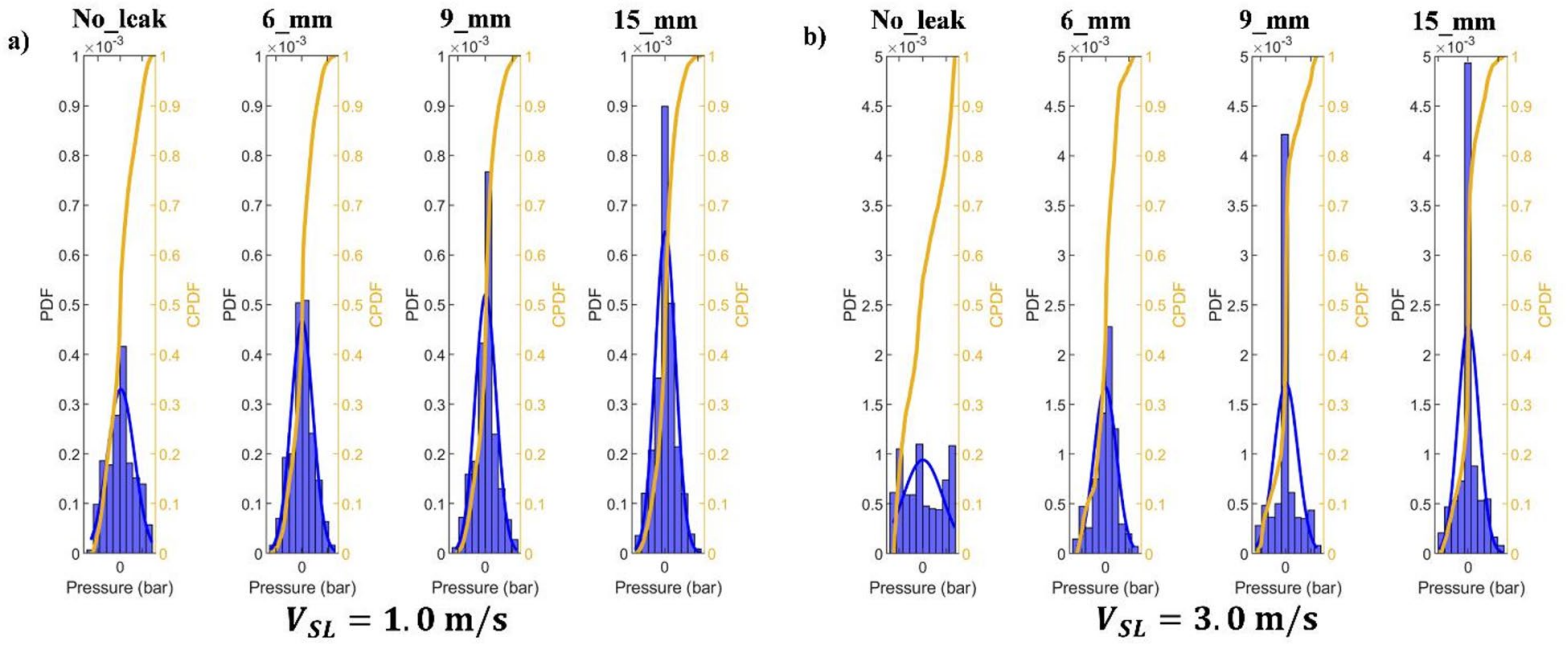
PDF and CPDF of the collected pressure fluctuation considering $\:{V}_{SG}$ of 0.4 m/s. (**a**) $\:{V}_{SL}=1\:m/s$. (**b**) $\:{V}_{SL}=3\:m/s$.

Figure 19a,b exhibit the PDF and CPDF of the collected pressure fluctuation considering $\:{V}_{SG}$ of 0.4 m/s. Under these operating conditions, it can be observed that the PDF shows a similar behavior for leak and no-leak cases, especially for 6 mm. In addition, when the discharge size increases from 6 to 15 mm, the PDF increments by 116% and 93%, respectively, for the for the $\:{V}_{SL}=1.0\:m/s$ and $\:{V}_{SL}=3.0\:m/s$. Which makes it less sensitive to identify discharge occurrence as compared to the case $\:{V}_{SG}$ of 0.1 m/s. Thus, it can be outlined that the PDF has more capabilities to identify the existence of discharge in pipelines transporting multiphase flows under low values of gas superficial velocity.

### Gas release nomograph

Targeting the aim of facilitating the determination of the release velocity of a leaky subsea pipeline transporting multiphase flow, a nomograph is developed based on a dimensionless analysis considering numerical data. After that, the developed nomograph results are compared with experimental data collected from the flow loop system. To perform that, the Buckingham π theorem is selected among other methods since it can provide information about dimensional groups and it has good scalability for high numbers of variables^44^. In addition, this method is widely applied to various phenomena of fluid flows^45,46^.

It is found that the release velocity of the gas phase in the water tank can be correlated with the gas void fraction in the main pipeline. Additionally, the latter is influenced by the operating conditions at the inlet, and it can be expressed by the mixture Froude number as follows^47^:

6 $$\:{Fr}_{M}=\frac{{V}_{M}}{\sqrt{g{D}_{P}}}\sqrt{\frac{{\rho\:}_{L}}{{\rho\:}_{L}-{\rho\:}_{G}}}$$

where $\:{V}_{M}$ stands for the mixture velocity and is expressed as $\:{V}_{M}={V}_{SG}+{V}_{SL}$, $\:{D}_{P}$ represents the diameter of the main pipeline, $\:g$ is the gravity, $\:{\rho\:}_{L}$ and $\:{\rho\:}_{G}$ are the density of the liquid and gas phases, respectively.

Based on that, it can be supposed that the gas volume fraction within the pipeline and gas release velocity are mainly influenced by release height, main pipeline diameter, discharge size, mass flow rate of both phases, gravity, and release velocity. These parameters can be expressed as:

7 $$\:Pipeline\:gas\:void\:fraction\:\left(\alpha\:\right)=f({H}_{o},{S}_{L},{\dot{m}}_{L},{\dot{m}}_{G},\:g,\:{V}_{R},{D}_{P})$$

8 $$\:Release\:velocity\:\left({V}_{R}\right)=f({H}_{o},{S}_{L},{\dot{m}}_{L},{\dot{m}}_{G},\:g,\:{V}_{R},{D}_{P})$$

Necessitating five dimensionless groups to comprehensively characterize a system comprising eight independent variables (see Table 4) with three distinct basic dimensions (mass M, length L, and time T) as basic quantities, whereas three parameters are considered to be redundant variables ($\:{H}_{R}$, $\:\dot{m}$, and $\:g$). According to the Buckingham-π theorem, the formulated dimensionless groups can be expressed in Table 5. In addition, the validity range of the considered parameters is highlighted in Table 6.

**Table 4:** Considered variables for dimensionless analysis.

Variable	Symbol	Units		
		L (m)	M (kg)	T (s)
Release height	$\:{H}_{R}$	1	0	0
Size of discharge	$\:{S}_{L}$	1	0	0
Pipeline gas void fraction	$\:\alpha\:$	0	0	0
Liquid flowrate	$\:{\dot{m}}_{L}$	0	1	− 1
Gas flowrate	$\:{\dot{m}}_{G}$	0	1	− 1
Acceleration of gravity	$\:g$	1	0	− 2
Release velocity	$\:{V}_{R}$	1	0	− 1
Pipeline diameter	$\:{D}_{P}$	1	0	0

**Table 5:** Developed dimensionless groups.

Dimensionless group number	Expression	Designation
$\:{{\varPi\:}}_{1}$	$\:\frac{{V}_{M}}{\sqrt{g{D}_{P}}}\sqrt{\frac{{\rho\:}_{L}}{{\rho\:}_{L}-{\rho\:}_{G}}}$	Froude number considering mixture velocity
$\:{{\varPi\:}}_{2}$	$\:\boldsymbol{\alpha\:}$	Gas void fraction within the main pipeline
$\:{{\varPi\:}}_{3}$	$\:\frac{{\boldsymbol{V}}_{\boldsymbol{R}}}{\sqrt{{\boldsymbol{g}\times\:\boldsymbol{H}}_{\boldsymbol{R}}}}$	Dimensionless release velocity
$\:{{\varPi\:}}_{4}$	$\:\frac{{\boldsymbol{H}}_{\boldsymbol{R}}}{{\boldsymbol{D}}_{\boldsymbol{P}}}$	Dimensionless release height
$\:{{\varPi\:}}_{5}$	$\:\frac{{\boldsymbol{S}}_{\boldsymbol{L}}}{{\boldsymbol{H}}_{\boldsymbol{R}}}$	Dimensionless discharge size

**Table 6:** Validity range of the considered parameters.

Parameter	Symbol	Range of validity
Superficial liquid velocity	$\:{V}_{SL}$	1–3 m/s
Superficial gas velocity	$\:{V}_{SG}$	0.1–0.4 m/s
Mixture velocity	$\:{V}_{M}$	1.1–3.4 m/s
Mixture Froude number	$\:{Fr}_{M}$	1.53–4.74
Release height	$\:{H}_{R}$	0.187–0.544 m
Discharge size	$\:{S}_{L}$	3–9 mm

#### Nomograph development

**Fig. 20:**
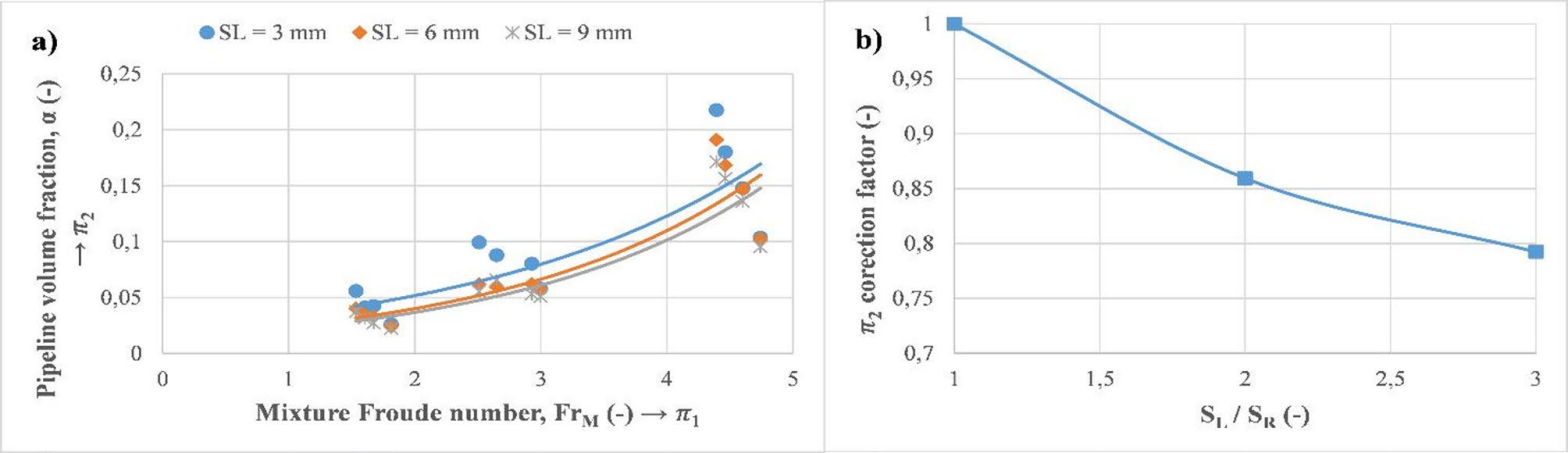
Representation of obtained dimensionless numbers. (**a**) Impact of the mixture Froude number ($\:{\varPi\:}_{1}$) on the pipeline void fraction ($\:{\varPi\:}_{2}$). (**b**) Correction factor of ($\:{\varPi\:}_{2}$) as a function of dimensionless discharge size.

Firstly, the impact of the mixture Froude number ($\:{\varPi\:}_{1}$) on the gas void fraction within the pipeline ($\:{\varPi\:}_{2}$) is plotted separately for various discharge sizes, as seen in Fig. 20a. In this Figure, the determined data are found to show an increasing trend where it is correlated by exponential law (based on the R^2^ score of data fitting). This trend can be explained by the fact that flow inertia dominates the external force field of the flow induced by the gas phase resulting in an increase of the pipeline volume fraction. Additionally, it can be observed that as the discharge size increases from 3 to 9 mm, the gas void fraction decreases by around 20%. To account for this effect, a correction factor is introduced to facilitate the determination of the latter (Fig. 20b).

**Fig. 21:**
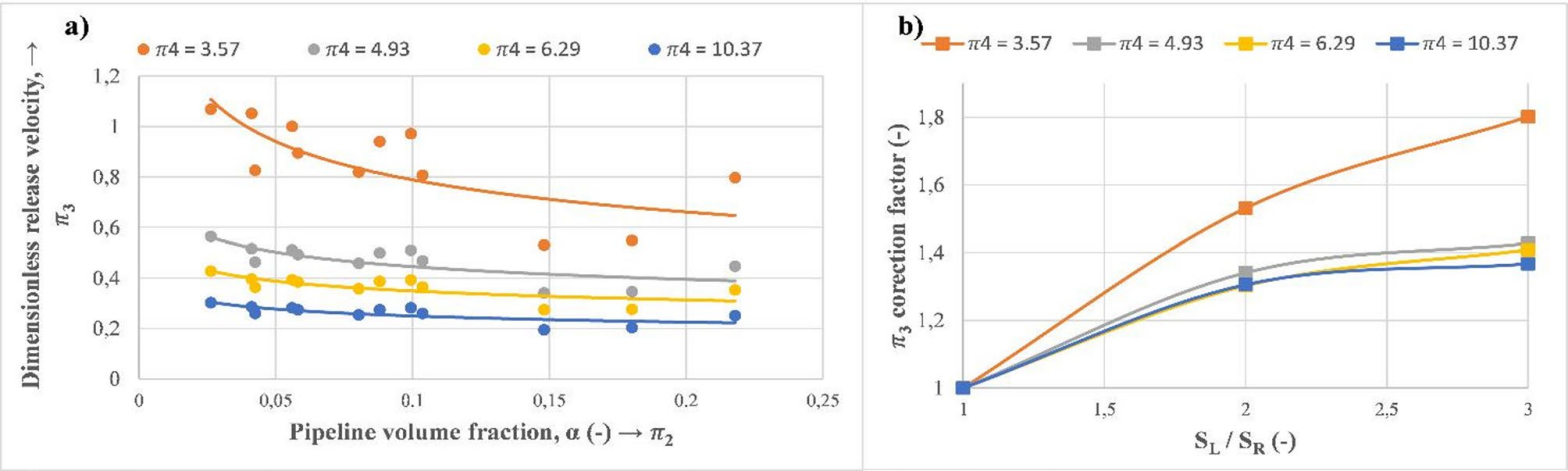
Representation of obtained dimensionless numbers. (**a**) Impact of the pipeline void fraction ($\:{\varPi\:}_{2}$) on the dimensionless release velocity ($\:{\varPi\:}_{3}$). (**b**) Correction factor of ($\:{\varPi\:}_{3}$) as a function of dimensionless discharge size.

Figure 21a exhibits variation of the dimensionless release velocity ($\:{\varPi\:}_{3}$) as a function of the pipeline void fraction ($\:{\varPi\:}_{2}$) for various values of the dimensionless release height ($\:{\varPi\:}_{4}$). This behavior is fitted with decreasing exponential variation, and it is more evident for the lowest value of $\:{\varPi\:}_{4}$. This effect diminishes rapidly to reach a stable value as the dimensionless release height ($\:{\varPi\:}_{4}$) increases (getting to higher levels from the main pipeline). In this case, as the discharge size increments from 3 to 9 mm, the dimensionless release velocity increases by around 40% and 80% for $\:{\varPi\:}_{4}=3.57$ and $\:{\varPi\:}_{4}=10.37$, respectively. Momentum reduction of the gas phase due to the hydrostatic pressure of water explains this behavior where it reaches a certain stable value after reaching a certain height^24^. Similarly, a correction factor is determined for $\:{\varPi\:}_{3}$ to facilitate its calculation for various discharge sizes (Fig. 21b).

**Fig. 22:**
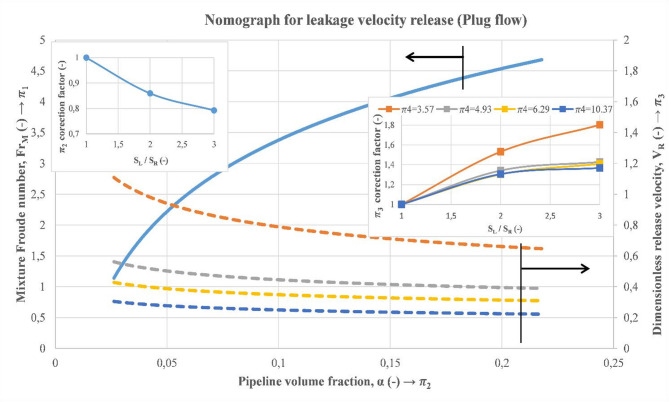
Nomograph for estimation of the gas release velocity of a leaky subsea pipeline transporting multiphase flow.

**Fig. 23:**
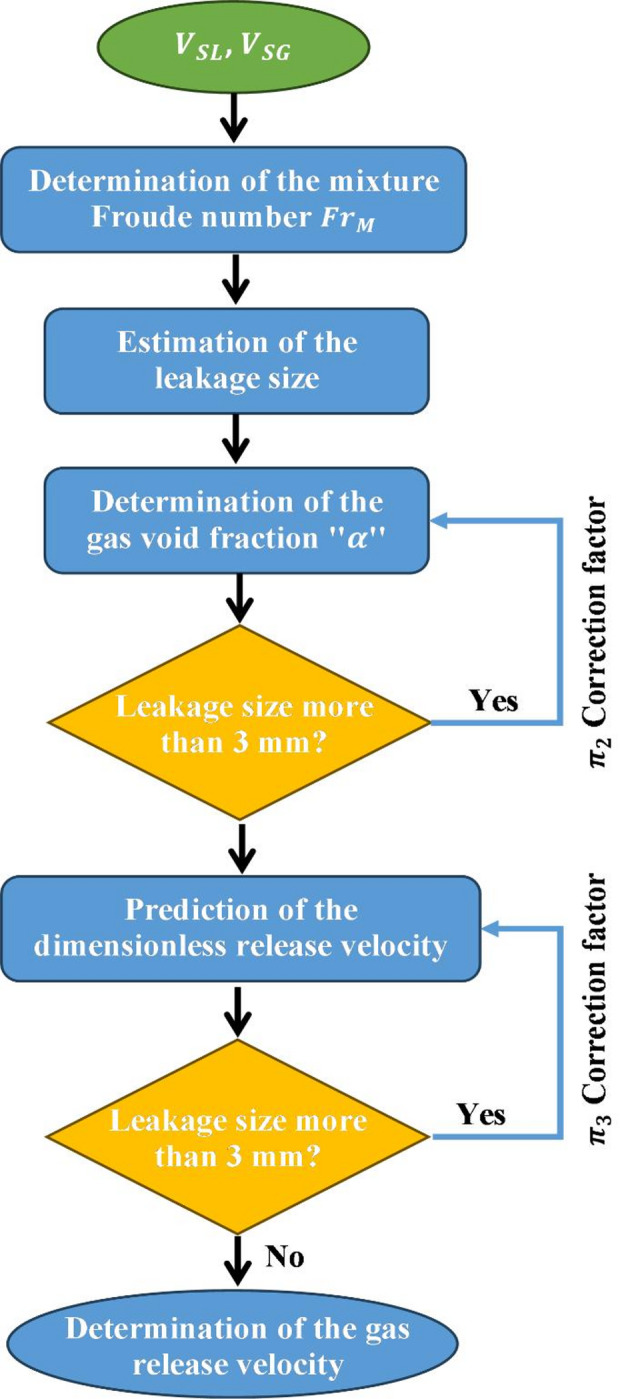
Flowchart of the calculation process of the gas release velocity.

Considering previous relationships between the dimensionless quantities $\:{\varPi\:}_{1}$, $\:{\varPi\:}_{2}$, $\:{\varPi\:}_{3}$, and $\:{\varPi\:}_{4}$, a nomograph is developed to determine the release velocity of a leaky subsea pipeline conveying two-phase flow, as can be seen in Fig. 22. To achieve this, an iterative process needs to be followed, starting from the calculation of the mixture Froude, the gas void fraction, and finally, the gas release velocity (Fig. 23).

The main steps to determine the release velocity can be summarized as follows:

1. Determination of the mixture Reynolds number.
2. Estimation of the discharge size based on the gas void fraction in the main pipeline (also, previous methods can be considered for the discharge size estimation based on pressure signals).
3. Correction of the gas void fraction estimated value if the discharge size is more than 3 mm.
4. After that, the dimensionless release velocity can be predicted at a specified height.
5. Correction of the dimensionless release velocity to account for the discharge size.
6. The gas release velocity can be determined by multiplying the obtained value times $\:\sqrt{{\boldsymbol{g}\times\:\boldsymbol{H}}_{\boldsymbol{R}}}$.

**Fig. 24:**
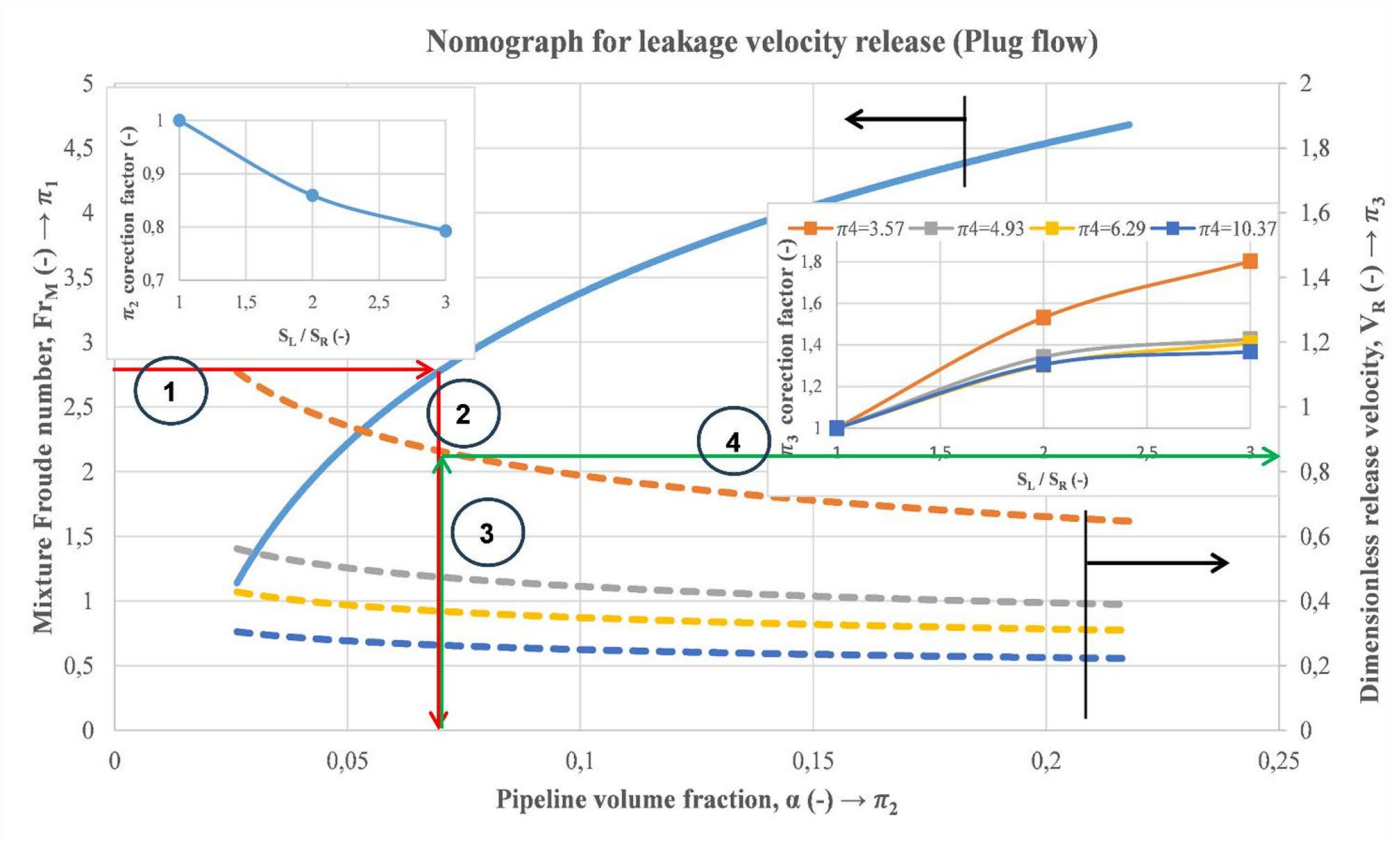
Illustrative example of the gas release velocity estimation at a certain release height considering the developed nomograph.

An example of the calculation steps of the gas release velocity for illustration as well as for validation with experimental data obtained from the experimental flow loop setup is shown below (Fig. 24). In this illustrative example, 2 cases were considered, as shown in Table 7. The first step consists of the estimation of the gas void fraction in the main pipeline using the blue continuous line. After that, the dimensionless release velocity is estimated based on the dashed lines, considering the suitable dimensionless release height. Subsequently, the release velocity is the result of the multiplication of the determined value times $\:\sqrt{{g\times\:H}_{R}}$. Results of the illustrated example and comparison with experimental data are exhibited below (Table 7). It is worth noting that in this example, the correction factors were not considered because the considered discharge size is 3 mm ($\:{S}_{L}/{S}_{R}=1$). In this table, the gas void fraction shows higher accuracies than the release velocity, and this can be accredited to the considered simplification in the discharge numerical model compared to the real case, as mentioned earlier. Therefore, the nomograph provides estimations with reasonable accuracy for the gas void fraction in the main pipeline as well as for the release velocity in the water tank.

**Table 7:** Considered cases and obtained results from the nomograph.

Superficial velocity (m/s)		Gas void fraction (–)		Release velocity (m/s)		Relative error (%)	
$\:{{V}}_{{S}{G}}$ (m/s)	$\:{{V}}_{{S}{L}}$ (m/s)	Nomograph	Experimental	Nomograph	Experimental	Gas void fraction	Release velocity
0.19	1.03	0.04	0.044	1.35	1.19	9	13.4
0.31	1.94	0.08	0.089	1.11	0.93	10.1	15.6

#### Limitations and future directions

The current analysis is limited to Laboratory scale conditions, including pipeline dimensions and operating conditions, which may limit the direct applicability of the results to complex industrial cases. However, the performed non-dimensional analysis could address this point by upscaling the fluid flow phenomena through non-dimensional numbers while the considered mechnaisms in this study are expected to remain valid. On the other hand, future directions will focus on extending the current work to account for the effect of high pressures and various types of fluid. This would be valuable for model generalization. Ultimately, integrating these developments could enable accurate predictive modeling under realistic conditions.

## Conclusions

The present investigation presents a comprehensive analysis of discharge (leakage) identification of a pipeline carrying gas-liquid multiphase flow (plug flow regime) considering transient numerical modeling of the flow within both pipeline and water tank to mimic under water conditions. After that, various methods were applied to the obtained time series signals, including statistical, probability density function, continuous wavelet transform, and dimensionless analysis to identify discharge occurrence in multiphase systems. Additionally, a nomograph was established to predict discharge release velocity in the case of underwater discharge occurrence. The findings can be summarized as follows:

1. Modeling of multiphase plug flow in a horizontal pipeline considering underwater discharge is successfully performed, where the numerical results were validated with experimental data obtained from an experimental setup.
2. Related pressure times series of multiphase flow phenomena in a horizontal pipeline with discharge may provide overall insights, however, additional analysis would be required to obtain meaningful information.
3. It was found that the fluctuation coefficient of the pressure drop signals intensifies with liquid and gas superficial velocities for the no-leak case, however, this effect is attenuated by the existence of discharge, where this effect decreases with its size.
4. In terms of the continuous wavelet transform, the frequency of the considered signals is mainly affected by the liquid superficial velocity, and its range is slightly influenced by the discharge size and number.
5. To estimate discharge dispersion of a leaky underwater pipeline carrying multiphase flow, a nomograph was developed to estimate discharge release velocity.

## Supplementary Information

Below is the link to the electronic supplementary material.

Supplementary Material 1

Supplementary Material 2

Supplementary Material 3

Supplementary Material 4

## Data Availability

The datasets generated and/or analysed during the current study are not publicly available because an additional analysis is required to generate them but they are available from the corresponding author on reasonable request.

## List of symbols

$$\:{D}_{P}$$: Pipeline diameter (m)
$\:{F}_{I}$: Two phase flow inertial forces (N)
$\:F$: Surface tension source term (N/m^3^)
$\:{F}_{V}$: Two phase flow viscous forces (N)
$\:{Fr}_{M}$: Mixture Froude number (–)
$\:g$: Acceleration of gravity (m/s^2^)
$$\:{H}_{R}$$: Release height (m)
$$\:I$$: Unit tensor
$\:{\dot{m}}_{L}$: Liquid flowrate (kg/s)
$\:{\dot{m}}_{G}$: Gas flowrate (kg/s)
$$\:P$$: Pressure (Pa)
$\:{Re}_{M}$: Mixture Reynolds number (–)
$\:{S}_{L}$: Size of leakage (m)
$\:{S}_{R}$: Size of reference leakage (3 mm)
$\:{S}_{\omega\:}$: Specific dissipation rate source term, (kg/m^3^ s^2^)
$$\:t$$: Time (s)
$$\:v$$: Velocity (m/s)
$\:V$: Pipeline total volume (m^3^)
$$\:{V}_{M}$$: Mixture velocity (m/s)
$$\:{V}_{R}$$: Release velocity (m/s)
$\:{V}_{SG}$: Gas superficial velocity (m/s)
$\:{V}_{SL}$: Liquid superficial velocity (m/s)
$\:\alpha\:$: Gas phase void fraction (–)
$\:{\alpha\:}_{Down}$: Gas phase void fraction downstream of the leakage (–)
$\:{\alpha\:}_{Up}$: Gas phase void fraction upstream of the leakage (–)
$\:k$: Kinetic energy of turbulence (m^2^/s^2^)
$\:{\mu\:}_{G}$: Gas viscosity (Pa s)
$\:{\mu\:}_{L}$: Liquid viscosity (Pa s)
$\:{\rho\:}_{G}$: Gas density (Pa s)
$\:{\rho\:}_{L}$: Liquid density (Pa s)
$\:\stackrel{-}{\tau\:}$: Molecular stress tensor (Pa)
$\:{\stackrel{-}{\tau\:}}_{t}$: Turbulent stress tensor
$\:{\mu\:}_{t}$: Turbulent viscosity (Pa s)
$\:\omega\:$: Specific dissipation rate (1/s)

## Abbreviations

CFD: Computational fluid dynamics
CPDF: Cumulative probability density function
CSF: Continuum surface force
CWT: Continuous wavelet transform
ERT: Electrical resistance tomography
PDF: Probability density function
PISO: Pressure-implicit with splitting of operators
PRESTO: PREssure STaggering Option
PSD: Power spectral density
RE: Relative error
RMS: Root mean square
SD: Standard deviation
SIMPLE: Semi-implicit method for pressure linked equations
SST: Shear stress transport
VOF: Volume of fluid
