# Supplementary material for: Analysis and nomograph development for a leaky pipeline carrying plug flow based on numerical modeling and experimental validation
Source: Sci Rep. 2026 Mar 4;16:12128. doi: 10.1038/s41598-026-36759-w (PMC13076652; doi:10.1038/s41598-026-36759-w)
Supplement: Supplementary file 1 — Supplementary Material 1 [file 41598_2026_36759_MOESM1_ESM.docx]

**Declaration of interests**

The authors declare that they have no known competing financial interests or personal relationships that could have appeared to influence the work reported in this paper.

The author is an Editorial Board Member/Editor-in-Chief/Associate Editor/Guest Editor for *[Journal name]* and was not involved in the editorial review or the decision to publish this article.

The authors declare the following financial interests/personal relationships which may be considered as potential competing interests:
