# Supplementary material for: Analysis and nomograph development for a leaky pipeline carrying plug flow based on numerical modeling and experimental validation
Source: Sci Rep. 2026 Mar 4;16:12128. doi: 10.1038/s41598-026-36759-w (PMC13076652; doi:10.1038/s41598-026-36759-w)
Supplement: Supplementary file 2 — Supplementary Material 2 [file 41598_2026_36759_MOESM2_ESM.docx]

- Numerical simulations of a pipeline carrying plug multiphase flow considering outside environment (water tank) to mimic under water conditions.
- Flow loop experimental setup was considered to validate numerical outputs against gathered experimental data.
- Wavelet analysis was applied for the obtained time series results of pressure output to identify leakage events.
- Non-dimensionless analysis was performed and a nomograph was developed to estimate gas release in the case of leakage events.
