# Supplementary material for: Analysis and nomograph development for a leaky pipeline carrying plug flow based on numerical modeling and experimental validation
Source: Sci Rep. 2026 Mar 4;16:12128. doi: 10.1038/s41598-026-36759-w (PMC13076652; doi:10.1038/s41598-026-36759-w)
Supplement: Supplementary file 4 — Supplementary Material 4 [file 41598_2026_36759_MOESM4_ESM.docx]

## Supplementary file (Governing equations)

The air-water multiphase flow within the flow domain is governed by the equations expressed below. Firstly, the flow phenomena is modeled using the Volume Of Fluid (VOF) where it is supposed to be isothermal, adiabatic, and incompressible. Equations (A1) and A2, respectively, describe the continuity and momentum of the multiphase flow via the VOF method which is considered as one fluid approach (Chinello et al., 2019*):

$\frac{\partial\rho}{\partial t}+\nabla.(\rho\vec{v})=0$ (A1)

$\frac{\partial\rho}{\partial t}\left( \rho\vec{v} \right)+\nabla.\left( \rho\vec{v}\vec{v} \right)=-\nabla p+\nabla.\left( \bar{\tau}+\bar{\tau}_{t} \right)+\rho\vec{g}+\vec{F}$ (A2)

where $\rho$ stands for the density of the mixture, $\vec{v}$ represents the vector of velocity, $p$ is pressure, $\vec{g}$ stands for the gravity acceleration, $\vec{F}$ is source term that accounts for surface tension effect which is modeled through the Continuum Surface Force (CSF) method (Brackbill1992). The tensor of molecular stress $\bar{\tau}$ is expressed as (Chinello et al., 2019*; Li et al., 2019**):

$\bar{\tau}=\mu\left[ \left( \nabla\vec{v}+\nabla\vec{v}^{T} \right)-\frac{2}{3}\left( \nabla.\vec{v}I \right) \right]$ (A3)

where $\vec{v}^{T}$ stands for the velocity vector transpose and $I$ represents the unit vector. The turbulent stress tensor (Reynolds stress) is estimated by the following relationship (Chinello et al., 2019*):

$\bar{\tau}_{t}=\mu_{t}\left[ \left( \nabla\vec{v}+\nabla\vec{v}^{T} \right)-\frac{2}{3}\left( \nabla.\vec{v}+\rho k \right)I \right]$ (A4)

The gas-liquid two-phase flow is treated as a mixture using the VOF concept, as per the studies already performed by (Lo and Tomasello, 2010) and Chinello et al. (2019)*. In this case, the density and viscosity of the mixture is evaluated based on gas volume fraction, as follows:

$\rho=\alpha_{L}\rho_{L}+\alpha_{G}\rho_{G}$ (A5)

$\mu=\alpha_{L}\mu_{L}+\alpha_{G}\mu_{G}$ (A6)

where $\alpha_{L}$ and $\alpha_{G}$ represent the primary (liquid) and secondary (gas) phases volume fraction, respectively, and related by the following expression:

$\alpha_{L}+\alpha_{G}=1$ (A7)

The volume fraction in each cell is determined by solving the following transport equation for one of the phases (the secondary phase is chosen):

$\frac{\partial\alpha_{G}}{\partial t}+\vec{v}.\nabla\alpha_{G}=0$ (A8)

The expression of the turbulent viscosity is given by (Chinello et al., 2019*):

$\mu_{t}=\frac{\rho k}{\omega}\frac{1}{max\left[ \frac{1}{\alpha^{*}},\frac{SF_{1}}{\alpha_{P}\omega} \right]}$ (A9)

where $k$ and $\omega$ are the turbulent kinetic energy and specific dissipation rate, respectively, and $S$ stands for the magnitude of strain rate and it is expressed as:

$S=\sqrt{2S_{ij}S_{ij}}$ (A10)

with $S_{ij}=\frac{1}{2}\left( \frac{\partial v_{i}}{\partial x_{j}}+\frac{\partial v_{j}}{\partial x_{i}} \right)$ which is the average of strain rate, $v_{i}$and $v_{j}$ represent the velocity components in $x_{i}$ and $x_{j}$, respectively. Then, the transport equations for $k$ and $\omega$ are written as:

$\frac{D\rho k}{Dt}=\frac{\partial}{\partial x_{j}}\left[ \left( \mu+\frac{\mu_{t}}{\sigma_{k}} \right)\frac{\partial k}{\partial x_{j}} \right]+\min\left( \mu_{t}S^{2}, 10\rho\beta^{*}k\omega\right)-\rho\beta^{*}k\omega$ (A11)

$\frac{D\rho\omega}{Dt}=\frac{\partial}{\partial x_{j}}\left[ \left( \mu+\frac{\mu_{t}}{\sigma_{\omega}} \right)\frac{\partial\omega}{\partial x_{j}} \right]+\frac{\alpha}{v_{t}}\min\left( \mu_{t}S^{2}, 10\rho\beta^{*}k\omega\right)-\rho\beta\omega^{2}+2\left( 1-F_{2} \right)\rho\frac{1}{\omega\sigma_{\omega,2}}\frac{\partial k}{\partial x_{j}}\frac{\partial\omega}{\partial x_{j}}+S_{\omega}$ (A12)

where the additional source term, $S_{\omega}$ is expressed as:

$S_{\omega}=A\Delta n\beta\rho\left( \frac{B6\mu}{\beta\rho\left( \Delta n \right)^{2}} \right)^{2}$ (A13)

where $\Delta n$ represents cell height normal to the interface, $B$ is a tuning parameter of turbulence damping, and $\beta$ a constant related to the turbulence model. Additionally, the blending functions $F_{1}$ and $F_{2}$ are written as:

$F_{1}=tanh\left[ max\left( \frac{2\sqrt{k}}{0.09\omega y}, \frac{500\mu}{\rho y^{2}\omega} \right) \right]^{2}$ (A14)

$F_{2}=tanh\left[ min\left[ max\left( \frac{\sqrt{k}}{0.09\omega y}, \frac{500\mu}{\rho y^{2}\omega} \right),\frac{4\rho k}{\sigma_{\omega,2}D_{\omega}^{+}y^{2}} \right] \right]^{4}$ (A15)

where $y$ stands for the closest distance to wall surface and $D_{\omega}^{+}$ represents a dimensionless specific dissipation rate. The constants related to the $k-\omega$ SST turbulence model and additional details are highlighted in the study of Chinello et al. (2019)*.

*Chinello, G., Ayati, A.A., McGlinchey, D., Ooms, G., Henkes, R., 2019. Comparison of computational fluid dynamics simulations and experiments for stratified air-water flows in pipes. J Fluids Eng 141, 051302.

**Li, X., Chen, G., Khan, F., Xu, C., 2019. Dynamic risk assessment of subsea pipelines leak using precursor data. Ocean Engineering 178, 156–169.
